# Trends in Weight Loss Attempts and Strategies Among US Adolescents With Overweight or Obesity

**DOI:** 10.1002/oby.70184

**Published:** 2026-04-05

**Authors:** Ligang Liu, Guang Xiong, Hao Zhao, Hekai Shi, Min Liang, Milap C. Nahata

**Affiliations:** ^1^ Institute of Therapeutic Innovations and Outcomes, College of Pharmacy The Ohio State University Columbus Ohio USA; ^2^ Department of Pharmacy Practice and Administration, College of Pharmacy Western University of Health Sciences Pomona California USA; ^3^ Department of Geriatric Endocrinology and Metabolism The First Affiliated Hospital of Guangxi Medical University Nanning Guangxi China; ^4^ Surgery Centre of Diabetes Mellitus Capital Medical University Affiliated Beijing Shijitan Hospital Beijing China; ^5^ Department of General Surgery, Huadong Hospital Fudan University Shanghai China; ^6^ College of Medicine The Ohio State University Columbus Ohio USA

**Keywords:** adolescent, NHANES, obesity, significant weight loss, trend, weight loss strategies

## Abstract

**Objective:**

This study assessed national trends in weight loss attempts, clinically meaningful weight loss, and associated strategies among US adolescents with overweight or obesity.

**Methods:**

We analyzed National Health and Nutrition Examination Survey (NHANES) 1999–2023 data for 2708 adolescents aged 16–19 years with overweight or obesity. Weight loss attempts and strategies were self‐reported. Clinically meaningful weight loss was defined as ≥ 5% and ≥ 10% body weight reductions or a ≥ 0.2 reduction in body mass index (BMI) *z*‐score over the prior year. Survey‐weighted logistic regression assessed temporal trends and associations between strategies and weight loss success.

**Results:**

The prevalence of weight loss attempts increased from 53.9% in 1999–2000 to 65.7% in 2021–2023 (*p* for trend < 0.001). Overall, 26.96% achieved a ≥ 5% weight loss and 12.58% achieved a ≥ 10% weight loss; 34.34% achieved a ≥ 0.2 BMI *z*‐score reduction. Exercise and dietary changes were the most reported strategies. Reducing sugar intake (OR, 2.16) and prescription medication use (OR, 2.55) were associated with achieving a ≥ 5% weight loss, and prescription medication use (OR, 3.59) was also associated with achieving a ≥ 10% weight loss. Sugar intake reduction (OR, 1.89) and prescription medication use (OR, 2.73) were associated with achieving a ≥ 0.2 reduction in BMI *z*‐score.

**Conclusions:**

Weight loss attempts increased over time, but clinically meaningful weight loss remained uncommon. Improved access to evidence‐based, developmentally appropriate interventions is needed.

## 1 Introduction

Adolescent obesity has emerged as a critical and escalating public health challenge as excess weight accrued during youth has strongly predicted the development of obesity, cardiometabolic disease, and psychosocial burden in adulthood [1]. In the United States (US), the prevalence of obesity among adolescents aged 12–19 years has risen dramatically over the past decades, now affecting approximately 21% of this population [2]. The economic burden of childhood overweight and obesity is substantial, contributing significantly to annual health care expenditures and long‐term societal costs [3, 4]. Beyond prevalence and cost, it is important to understand how adolescents attempt to lose weight and whether those attempts lead to clinically meaningful weight reduction because weight control behaviors can shape physical health and are also linked to eating pathology risk and mental well‐being [5, 6].

Between 2013 and 2016, an estimated 58.9% of US adolescents aged 16–19 years with overweight and 77.7% with obesity reported trying to lose weight [7]. To achieve weight loss, adolescents used a wide range of weight control strategies [8]. These behaviors spanned healthy weight control behaviors (HWCBs), such as increasing physical activity and improving diet, which have been associated with favorable health outcomes [9], as well as unhealthy weight control behaviors (UWCBs), such as fasting, skipping meals, or using diet pills, which have been linked to adverse physical and psychological consequences [10, 11, 12]. In national data from 1999 to 2013, the prevalence of UWCBs was 16.64% [13]. Adolescents with overweight or obesity are more likely to report engaging in both HWCBs and UWCBs than their healthy‐weight peers [14]. However, the extent to which these reported efforts translate into meaningful weight reduction remains unclear. In an analysis of National Health and Nutrition Examination Survey (NHANES) 2001–2006 data, approximately 20% of US adults with obesity achieved ≥ 10% weight loss and about 40% achieved ≥ 5% weight loss among those who reported attempting to lose weight [15].

Despite these observations, key evidence gaps remain. First, national temporal trends in achieving clinically significant weight loss (e.g., ≥ 5% or ≥ 10% of body weight) among adolescents with overweight or obesity are not well characterized. Second, there is limited understanding of which self‐reported strategies are most strongly associated with meaningful weight loss success at the population level. Third, few studies have examined differences across demographic and socioeconomic groups in both weight loss efforts and outcomes using consistent definitions over an extended period.

The need to close these gaps in evidence is particularly timely because the social and clinical context of adolescent weight management has changed markedly over the past two decades [16]. Adolescents are increasingly exposed to evolving social media influences and diet trends, and clinical practice has expanded access to evidence‐based interventions, including structured behavioral programs and, more recently, obesity pharmacotherapy in pediatric populations [17]. These shifts may affect both the prevalence of weight loss attempts and the strategies used, potentially altering the likelihood of achieving meaningful weight loss.

Therefore, by using nationally representative NHANES data from 1999 to 2023, we examined trends in weight loss attempts, weight loss strategies, and achievement of clinically significant weight loss among adolescents aged 16–19 years. We restricted analyses to adolescents aged 16–19 years because weight loss‐related questionnaire items were available and could be harmonized consistently across NHANES cycles in this age group. This range also represented a more developmentally comparable period of late adolescence with greater autonomy over diet, physical activity, and health care decisions. We hypothesized that weight loss attempts would increase over time, but that the prevalence of clinically meaningful weight loss would remain low, and that evidence‐based strategies would be strongly associated with weight loss success.

## 2 Methods

### 2.1 Study Design and Population

This cross‐sectional study utilized repeated survey data from NHANES from 1999–2000 through 2021–2023. NHANES provides a nationally representative sample of the US population through a combination of interviews and physical examinations [18]. The interviews collected demographic, socioeconomic, dietary, and health information, and the examinations included medical, dental, laboratory, and physiological assessments. This study followed the Strengthening the Reporting of Observational Studies in Epidemiology (STROBE) reporting guideline [19]. The Ohio State University Institutional Review Board determined this study was exempt from review and informed consent, as it utilized publicly available deidentified data.

### 2.2 Study Population

**FIGURE 1:**
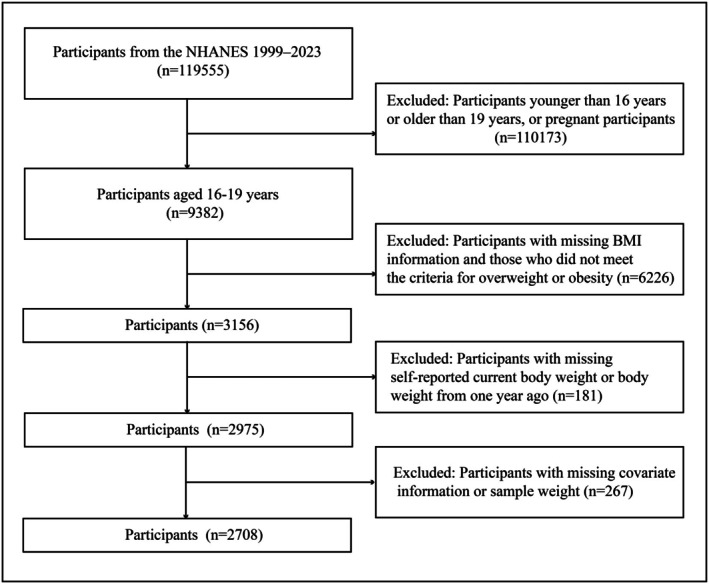
Flowchart of participant selection in the study.

The study included adolescents aged 16–19 years with overweight or obesity, defined using body mass index (BMI) percentiles based on the sex‐specific Centers for Disease Control and Prevention (CDC) 2000 growth charts. Overweight was defined as BMI in the 85th to < 95th percentile, and obesity as BMI ≥ 95th percentile [17]. Among 119,555 NHANES participants from 1999 to 2023, we excluded 110,173 participants who were outside the 16– to 19‐year age range or pregnant. We further excluded 6226 participants with missing BMI data or who did not meet predefined criteria for overweight or obesity, 181 participants with missing body weight, and 267 participants missing covariates or sample weights. The final analytic sample included 2708 adolescents (Figure 1).

### 2.3 Quality Control

Data pertaining to body weight history were obtained from the Body Weight History Questionnaire module. Trained interviewers conducted standardized in‐home interviews using the Computer‐Assisted Personal Interview (CAPI) system for participants aged ≥ 16 years. Information collected included self‐reported current and prior‐year body weight, perceived weight status, weight loss attempts during the past year, and weight loss strategies used. The CAPI system was equipped with automated consistency checks to reduce data entry errors and included an integrated help function that standardized definitions and interviewer guidance, supporting uniform data collection across interviewers, geographic regions, and survey cycles.

### 2.4 Weight Loss Attempts

Participants were classified as having attempted weight loss based on responses to NHANES weight history questions. First, participants who reported that their current weight was at least 10 lb lower than their weight 1 year earlier were asked whether this weight change was intentional; those who answered “yes” were classified as having attempted weight loss. Second, all participants were asked directly whether they had tried to lose weight during the past 12 months; respondents who answered “yes” were also classified as having attempted weight loss. Participants who met either criterion were categorized as having attempted weight loss.

### 2.5 Achievement of Weight Loss

Participants reported their current body weight and weight from 1 year ago. Weight from a year prior served as the initial weight. The percentage of weight loss was calculated as:

$$\begin{array}{c} \text{Weight loss percentage}=\left(\text{Initial weight}-\text{Current weight}\right)\\ \;÷\text{Initial weight}\times 100 \end{array}$$

Participants achieving reductions of ≥ 5% or ≥ 10% were classified as having achieved significant weight loss [20, 21]. As prior‐year height was not available in NHANES, we estimated it by deducting age‐ and sex‐specific annual height growth velocity from current height [22]. This estimated prior‐year height was used to calculate initial BMI with self‐reported prior‐year weight. BMI *z*‐scores were derived from the 2000 CDC sex‐ and age‐specific BMI‐for‐age growth charts, with BMI *z*‐score reduction (initial BMI *z*‐score minus current BMI *z*‐score) ≥ 0.20 also defined as significant weight loss [23].

### 2.6 Weight Loss Strategies

Participants who attempted to lose weight were asked to report the strategies they used. Across NHANES cycles from 1999–2000 to 2017–March 2020, the questionnaire included 14–21 weight loss strategies, depending on the survey cycle. The survey cycle from August 2021 to August 2023 did not collect information on specific weight loss strategies. Fourteen options were consistently available across all survey cycles: (1) reduced food intake, (2) switched to lower‐calorie foods, (3) reduced fat intake, (4) exercised, (5) skipped meals, (6) used diet foods or products, (7) used a liquid diet formula, (8) participated in a weight loss program, (9) took prescription medications, (10) used nonprescription dietary supplements, (11) used laxatives or induced vomiting, (12) drank large amounts of water, (13) followed a special diet, or (14) other methods. Starting in 2005–2006, five additional options were introduced: (1) reduced carbohydrate intake, (2) started or resumed smoking, (3) increased consumption of fruits, vegetables, and salads, (4) changed eating habits, and (5) reduced intake of sugar, candy, and sweets. Reduced junk/fast food consumption was added from 2009‐2010 onwards. Weight loss surgery was introduced starting in 2013‐2014. Participants could select multiple strategies, and responses were not mutually exclusive.

### 2.7 Covariates

Covariates included age, sex, race/ethnicity (non‐Hispanic White, non‐Hispanic Black, Mexican American, other races), and family income. Family income was categorized using the poverty income ratio (PIR) into three levels: low income (PIR < 1.3), middle income (1.3 ≤ PIR < 3.5), and high income (PIR ≥ 3.5).

### 2.8 Statistical Analysis

Considering the complex sampling design of NHANES, all analyses incorporated appropriate stratification, clustering, and sample weighting to ensure nationally representative estimates. Descriptive statistics were used to summarize participant demographics and weight loss behaviors. Continuous variables were presented as weighted means with standard errors, while categorical variables were displayed as unweighted frequencies and weighted percentages. Adjusted rates/proportions were standardized for age using the 2000 US Census population for adolescents aged 16–19 years.

Weighted logistic regression models were applied to assess the temporal trends in weight loss attempts, achievement of clinically significant weight loss, and weight loss strategies, with the midpoint of each survey cycle included as a continuous independent variable in the models. Unadjusted models and adjusted models controlling for age, sex, race/ethnicity, and family income were constructed separately. The average annual percent change (AAPC) was calculated to quantify the annual trend over the study period.

Multivariable logistic regression models were established to analyze the association between weight loss strategies and clinically significant weight loss outcomes. Standard multivariable logistic regression and the backward elimination method were used for model construction, with the final backward elimination model selected based on the minimum Akaike information criterion (AIC) value. Both modeling methods were used to fit unadjusted models and adjusted models controlling for age, sex, race, and family income, respectively.

The Taylor series linearization method was used for variance estimation. All multivariable regression models were tested using the variance inflation factor (VIF), with all variables showing a VIF value < 5, which reduced the potential for multicollinearity and ensured the robustness of the model estimates. A series of sensitivity analyses were conducted in this study: (1) constructing unadjusted models and adjusted models; (2) cross‐validating the trend test results of weighted logistic regression with the quantitative results of AAPC to enhance the reliability of conclusions regarding temporal trends; and (3) adopting cross‐validation with multiple outcomes and models and constructing standard multivariable logistic regression and backward elimination models for each outcome based on the multiple criteria for clinically significant weight loss (weight loss ≥ 5% and ≥ 10%, reduction in BMI *z*‐score ≥ 0.2) to verify the stability of association analysis results. AAPC was computed using the Joinpoint Trend Analysis Software (version 5.3.0), and all other analyses were conducted in R (version 4.4.1). Statistical significance was defined as a two‐sided *p* value < 0.05.

## 3 Results

Data were collected from 2708 participants with overweight or obesity between 1999 and 2023 (Figure 1). Of all participants (aged 17.37 ± 0.03 years), 1327 (47.44%) were girls, and 709 (53.91%) were White. Age, sex, race, and family income level did not differ significantly between these groups (Table 1). Characteristics of participants by survey year are presented in Table S1.

**TABLE 1:** Baseline characteristics of participants by BMI categories.

Characteristics	NHANES participants, *N* (weighted %)			*p*
	Total (*N* = 2708)	Obesity (*N* = 1479)	Overweight (*N* = 1229)	
Age (years)	17.37 ± 0.03	17.37 ± 0.04	17.37 ± 0.04	0.96
Sex				0.01
Boy	1381 (52.56)	792 (55.93)	589 (48.63)	
Girl	1327 (47.44)	687 (44.07)	640 (51.37)	
Race				0.09
Non‐Hispanic White	709 (53.91)	378 (52.73)	331 (55.29)	
Non‐Hispanic Black	812 (15.92)	487 (17.82)	325 (13.71)	
Mexican American	784 (15.45)	409 (15.55)	375 (15.34)	
Others	403 (14.72)	205 (13.91)	198 (15.67)	
Family income				0.42
Low income	1230 (35.25)	694 (36.61)	536 (33.67)	
Middle income	997 (38.37)	534 (36.91)	463 (40.08)	
High income	481 (26.37)	251 (26.48)	230 (26.25)	
BMI (kg/m^2^)	31.63 ± 0.16	35.33 ± 0.19	27.32 ± 0.06	< 0.01
Initial weight (lb)	188.74 ± 1.09	208.90 ± 1.46	165.20 ± 1.13	< 0.01
Current weight (lb)	194.27 ± 1.13	216.15 ± 1.38	168.75 ± 0.95	< 0.01
Weight change (lb)	−5.54 ± 0.53	−7.25 ± 0.81	−3.55 ± 0.66	< 0.01
Tried to lose weight				< 0.01
No	1068 (38.72)	503 (32.31)	565 (46.20)	
Yes	1640 (61.28)	976 (67.69)	664 (53.80)	

*Note*: Continuous variables are expressed as the weighted mean ± standard error; categorical variables are expressed as unweighted numbers (weighted percentages).

Abbreviation: NHANES, National Health and Nutrition Examination Survey.

### 3.1 Trends in Weight Loss Attempts

**FIGURE 2:**
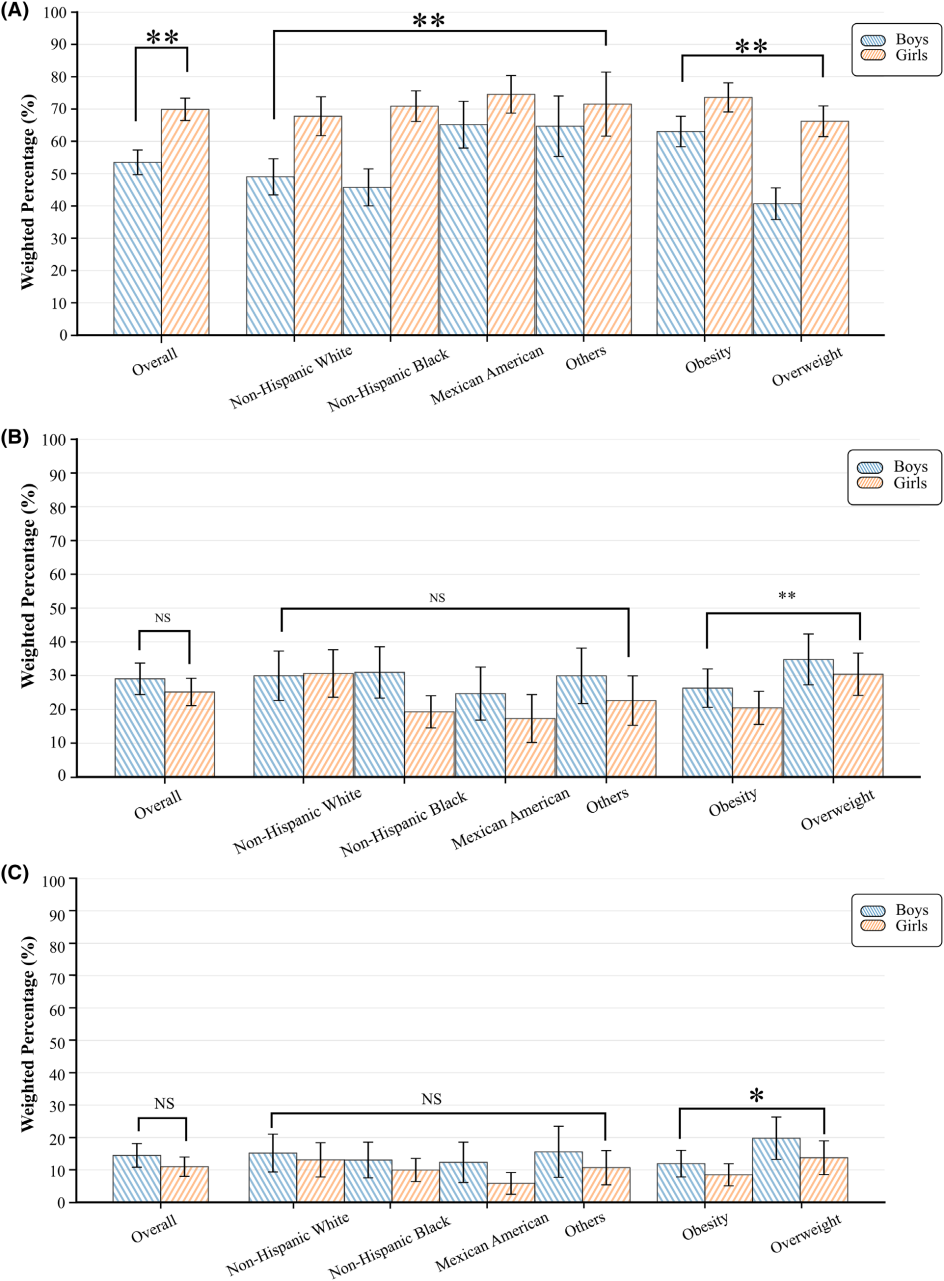
Weight loss attempts and achievement of clinically significant weight loss among US adolescents with overweight or obesity, stratified by sex, race/ethnicity, and weight status. (A) Percentage of participants reporting an attempt to lose weight. (B) Percentage achieving ≥ 5% weight loss among participants attempting to lose weight. (C) Percentage achieving ≥ 10% weight loss among participants attempting to lose weight. *p < 0.05, **p < 0.01; NS, not significant. [Color figure can be viewed at wileyonlinelibrary.com]

Overall, 61.28% of participants reported trying to lose weight, with a significantly higher proportion of girls (69.91%) compared to boys (53.5%) (*p* < 0.01). Significant racial differences were observed, with Mexican Americans (69.53%) and other races (67.78%) reporting higher weight loss attempts compared to White (57.60%) and Black participants (59.72%) (*p* < 0.01). Participants with obesity were more likely to report attempts to lose weight than those with overweight (Figure 2A, Table S2).

The weighted prevalence of participants attempting to lose weight increased significantly from 53.88% in 1999–2000 to 65.70% in 2021–2023 (AAPC, 1.08; 95% CI, 0.21 to 2.10; *p* for trend < 0.01). The prevalence of weight loss attempts increased among boys with overweight (21.75% to 48.79%), whereas the trend decreased among girls with overweight (73.01% to 59.96%). However, these changes were not significant. Among boys with obesity, the prevalence of weight loss attempts rose from 53.28% to 75.43% (AAPC, 1.87; 95% CI, 0.64 to 3.79; *p* for trend < 0.01). A small decrease in weight loss attempts was observed among girls with obesity (74.43% to 59.52%; *p* for trend = 0.73) (Table 2).

**TABLE 2:** Trends in past‐year weight loss attempts (%) among adolescents aged 16–19 years from 1999 to 2023: overall and stratified by sex and weight status.

Participation rates	1999–2000	2001–2002	2003–2004	2005–2006	2007–2008	2009–2010	2011–2012	2013–2014	2015–2016	2017–March 2020	August 2021–August 2023	*p* for trend	Average annual percent change (95% CI)
All participants													
Unadjusted	53.26 ± 4.41	57.52 ± 3.21	60.85 ± 2.50	59.86 ± 4.62	55.71 ± 5.68	43.68 ± 4.48	57.70 ± 5.10	66.63 ± 5.18	71.85 ± 3.19	68.18 ± 3.35	65.68 ± 4.20	< 0.01	1.01 (0.06 to 2.02)
Adjusted	53.88 ± 4.10	56.99 ± 3.19	60.86 ± 2.98	59.44 ± 4.73	55.92 ± 5.44	43.58 ± 4.31	57.73 ± 5.15	67.76 ± 4.96	72.58 ± 3.22	68.79 ± 3.20	65.70 ± 3.77	< 0.01	1.08 (0.21 to 2.10)
Boys, overweight													
Unadjusted	24.43 ± 4.31	52.33 ± 9.15	52.54 ± 5.75	39.14 ± 6.75	31.82 ± 8.57	17.24 ± 5.49	31.68 ± 7.10	42.54 ± 7.39	52.49 ± 8.81	47.59 ± 7.23	51.31 ± 12.60	0.21	0.93 (−3.18 to 4.44)
Adjusted	21.75 ± 4.59	50.26 ± 8.04	51.89 ± 5.79	39.87 ± 7.03	31.66 ± 8.45	19.04 ± 6.56	39.54 ± 7.63	41.17 ± 7.81	46.05 ± 9.78	46.05 ± 6.82	48.79 ± 12.25	0.21	0.53 (−2.08 to 2.82)
Boys, obesity													
Unadjusted	52.48 ± 7.86	37.06 ± 5.28	62.96 ± 7.60	47.76 ± 8.97	48.05 ± 9.56	48.37 ± 6.89	68.93 ± 5.92	87.23 ± 6.03	74.36 ± 6.84	76.11 ± 5.59	75.74 ± 5.95	< 0.01	2.44 (0.86 to 4.89)
Adjusted	53.28 ± 6.94	36.25 ± 5.76	65.75 ± 5.17	48.60 ± 8.06	48.01 ± 9.43	47.66 ± 7.28	62.93 ± 5.79	85.24 ± 5.01	73.13 ± 6.12	75.35 ± 5.11	75.43 ± 4.82	< 0.01	1.87 (0.64 to 3.79)
Girls, overweight													
Unadjusted	74.29 ± 4.33	80.11 ± 5.06	57.50 ± 7.62	74.17 ± 6.18	73.47 ± 11.60	43.11 ± 8.20	66.27 ± 5.36	64.04 ± 7.92	67.29 ± 8.70	63.46 ± 6.01	65.22 ± 9.18	0.22	−0.97 (−2.10 to −0.18)
Adjusted	73.01 ± 5.75	83.54 ± 4.31	57.71 ± 7.63	74.93 ± 5.68	76.48 ± 9.88	43.34 ± 7.52	63.16 ± 5.94	64.05 ± 7.68	70.13 ± 6.71	62.28 ± 7.09	59.96 ± 6.93	0.16	−1.30 (−2.79 to −0.33)
Girls, obesity													
Unadjusted	75.33 ± 7.46	68.46 ± 7.29	71.46 ± 6.15	79.20 ± 5.91	77.95 ± 6.98	62.22 ± 10.04	61.27 ± 8.37	68.20 ± 11.58	93.09 ± 3.37	81.17 ± 4.95	61.41 ± 6.30	0.72	−0.33 (−2.10 to 2.03)
Adjusted	74.43 ± 7.37	67.18 ± 7.10	70.36 ± 6.53	79.60 ± 6.19	80.85 ± 5.27	61.02 ± 8.23	60.88 ± 8.38	69.50 ± 8.53	92.55 ± 3.92	80.79 ± 4.92	59.52 ± 6.72	0.73	−0.37 (−2.89 to 3.50)

*Note*: Participation rates are presented as weighted percentage ± standard error. Adjusted rates are age‐standardized using the 2000 US Census population for adolescents aged 16–19 years. In the adjusted model, *p* for trend was additionally adjusted for age, sex, race/ethnicity, and family income. The average annual percent change is expressed as “estimate (95% CI)” to represent the average yearly change over the study period.

### 3.2 Achieving ≥ 5% Weight Loss

Overall, 26.96% of participants reported achieving ≥ 5% weight loss, with no significant differences between sexes or races. However, participants with overweight were more likely to achieve this target compared to those with obesity (Figure 2B, Table S3).

**FIGURE 3:**
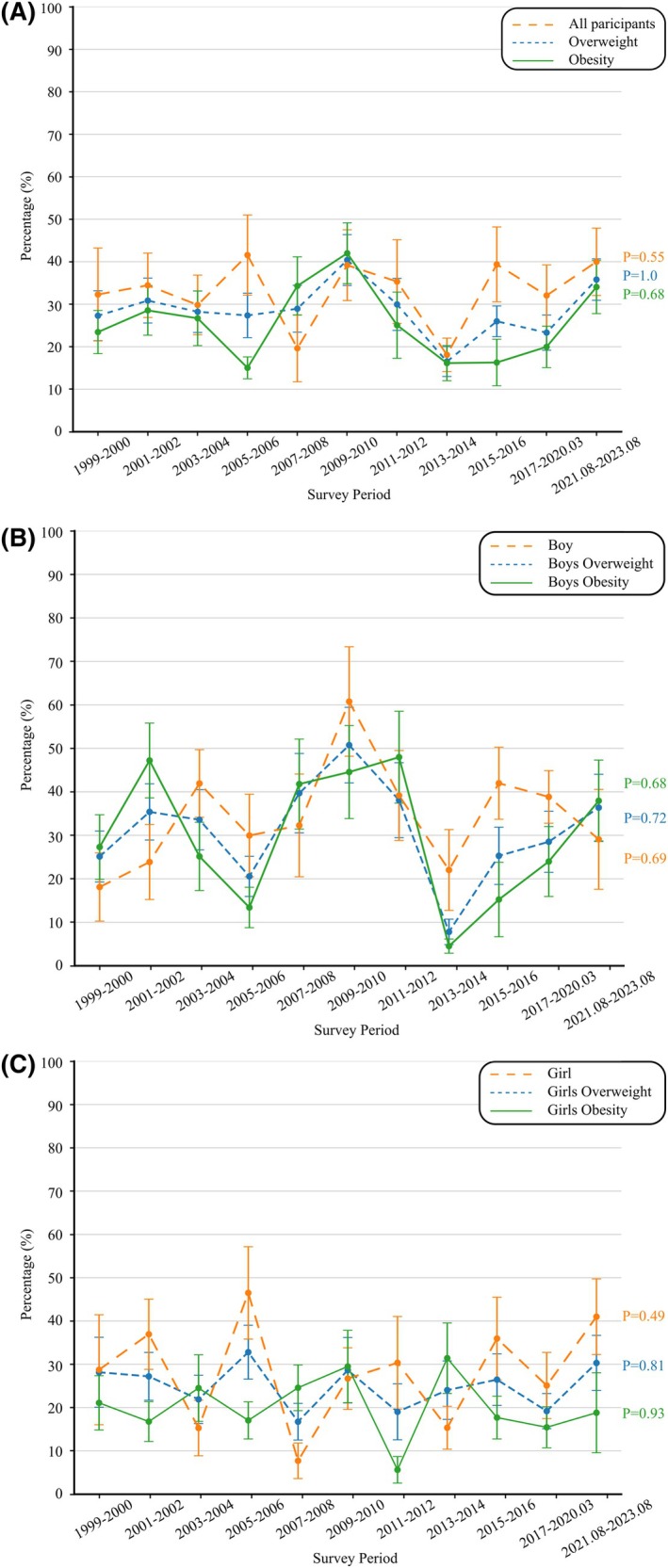
Trends in achieving ≥ 5% weight loss among US adolescents aged 16–19 years who reported attempting weight loss, NHANES 1999–2023. (A) Overall and stratified by weight status (overweight vs. obesity). (B) Boys (overall and by weight status). (C) Girls (overall and by weight status). [Color figure can be viewed at wileyonlinelibrary.com]

The weighted prevalence of participants achieving ≥ 5% weight loss did not change significantly over time (27.30% in 1999–2000 and 35.80% in 2021–2023; AAPC, 0.05; 95% CI, −1.93 to 2.25; *p* for trend = 1.00). Similar trends were observed for participants with obesity (AAPC, 0.61; 95% CI, −4.78 to 5.31; *p* for trend = 0.68) and those with overweight (AAPC, 0.25; 95% CI, −2.45 to 3.23; *p* for trend = 0.55). Among boys with overweight, the prevalence of achieving ≥ 5% weight loss increased from 18.11% to 29.08% (AAPC, 0.72; 95% CI, −2.92 to 5.33; *p* for trend = 0.69); it also increased among girls with overweight (28.75% to 41.00%; AAPC, 0.07; 95% CI, −4.17 to 4.61; *p* for trend = 0.49). Among boys with obesity, the prevalence increased from 27.31% to 37.94% (AAPC, −1.08; 95% CI, −10.90 to 7.82; *p* for trend = 0.68), but among girls with obesity, it declined from 21.10% to 18.82% (AAPC, −0.26; 95% CI, −4.95 to 4.08; *p* for trend = 0.93) (Figure 3A–C, Table S4).

### 3.3 Achieving ≥ 10% Weight Loss

Overall, 12.58% of participants achieved ≥ 10% weight loss, with no differences observed by gender, race, or BMI (Figure 2C, Table S5). The prevalence of achieving ≥ 10% weight loss did not increase significantly from 1999–2000 to 2021–2023 (AAPC, −0.51; 95% CI, −4.44 to 2.97; *p* for trend = 0.61). Moreover, no significant trends were observed for participants with obesity (6.85% to 14.49%; AAPC, −0.73; 95% CI, −5.86 to 3.59; *p* for trend = 0.84) or those with overweight (22.77% to 23.82%; AAPC, −0.29; 95% CI, −5.01 to 5.16; *p* for trend = 0.68) (Table S6).

**FIGURE 4:**
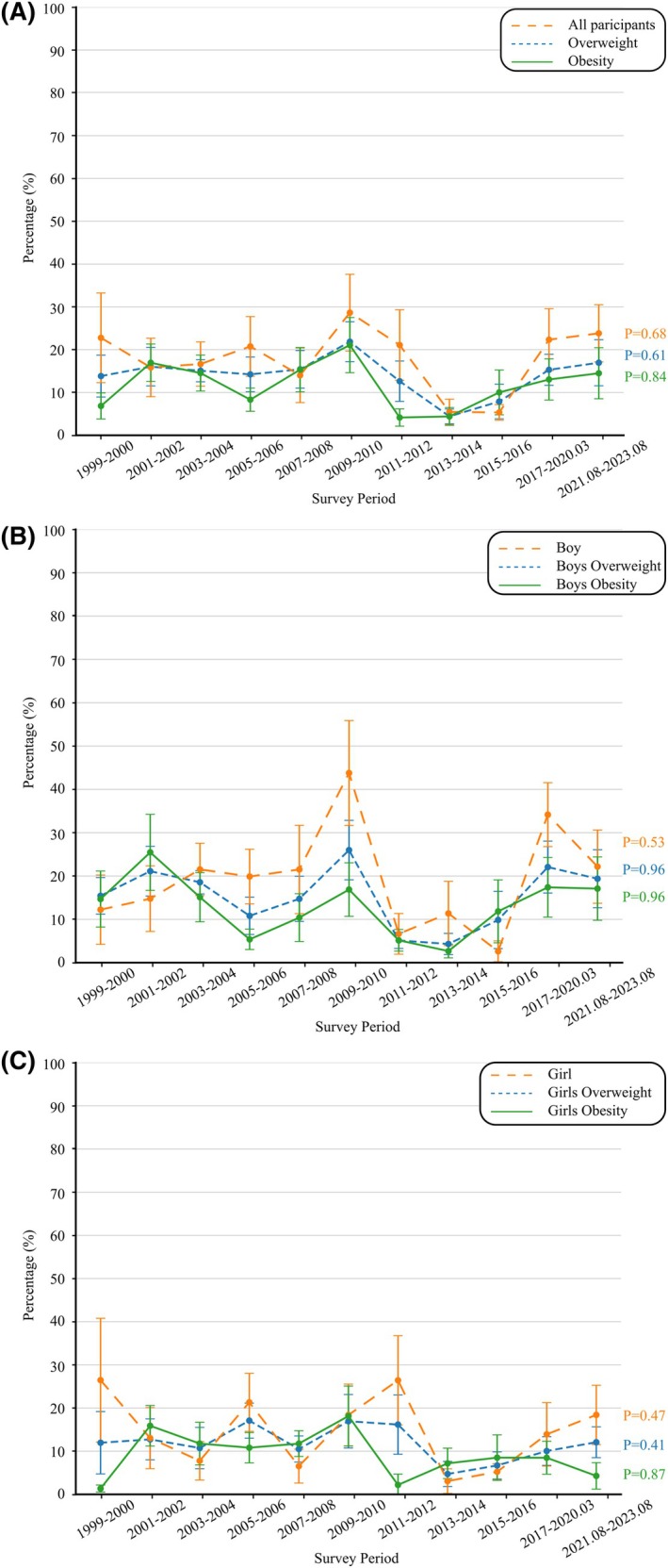
Trends in achieving ≥ 10% weight loss among US adolescents aged 16–19 years who reported attempting weight loss, NHANES 1999–2023. (A) Overall and stratified by weight status (overweight vs. obesity). (B) Boys (overall and by weight status). (C) Girls (overall and by weight status). [Color figure can be viewed at wileyonlinelibrary.com]

When stratified by gender and BMI category, boys with overweight experienced an increase in the prevalence of achieving ≥ 10% weight loss, rising from 12.23% to 22.16% (AAPC, 2.07; 95% CI, −2.70 to 8.14; *p* for trend = 0.53). In contrast, girls with overweight showed a decline from 26.46% to 18.41% (AAPC, −1.66; 95% CI, −9.55 to 6.77; *p* for trend = 0.47). Among boys with obesity, the prevalence of achieving ≥ 10% weight loss increased from 14.68% to 17.09% (AAPC, −1.07; 95% CI, −8.49 to 5.88; *p* for trend = 0.96). Similarly, among girls with obesity, this prevalence rose from 1.35% to 4.28% (AAPC, −1.71; 95% CI, −9.72 to 4.54; *p* for trend = 0.87) (Figure 4A–C, Table S6).

### 3.4 Weight Loss Strategies

**FIGURE 5:**
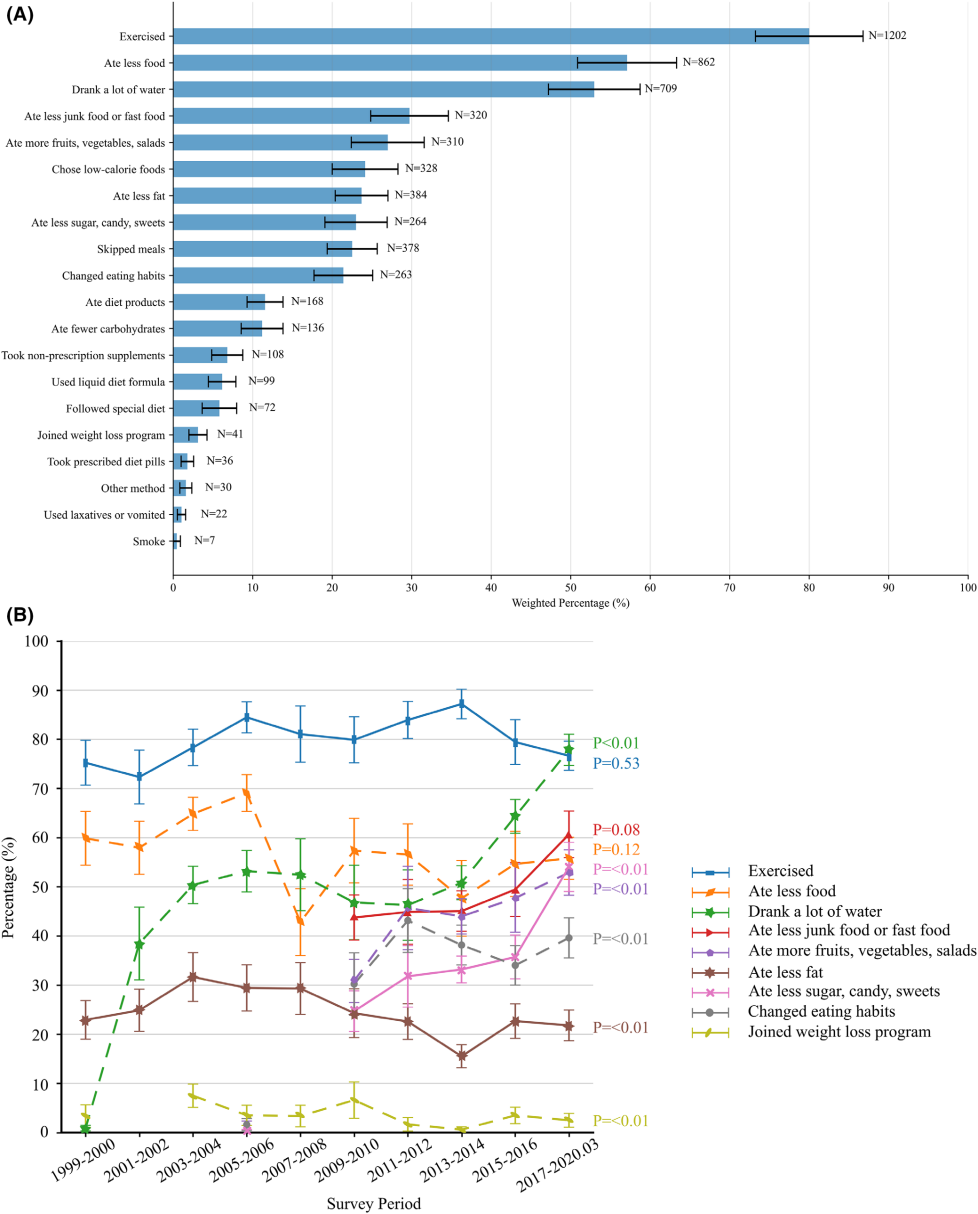
Weight loss strategies reported by US adolescents aged 16–19 years with overweight or obesity who attempted to lose weight, NHANES 1999–2020. (A) Distribution of reported weight loss strategies among adolescents attempting to lose weight. (B) Trends in selected weight loss strategies across NHANES survey cycles. [Color figure can be viewed at wileyonlinelibrary.com]

Exercise was the most reported weight loss strategy (80.01%), followed by eating less food (57.09%), drinking water (52.96%), and reducing junk food consumption (29.72%). Other dietary changes included consuming more fruits and vegetables (26.99%), choosing low‐calorie foods (24.14%), eating less fat (23.69%), and skipping meals (22.51%). Less common strategies included prescription medications (1.78%) and participating in weight loss programs (3.11%) (Figure 5A, Table S7). No participants reported undergoing weight loss surgery. From 1999–2000 to 2017–2020, eating more fruits, vegetables, and salads (30.87% to 52.91%, *p* for trend < 0.01), consuming less sugar (24.70% to 54.05%, *p* for trend < 0.01), changing eating habits (30.23% to 39.62%, *p* for trend < 0.01), and drinking water (0.92% to 77.88%, *p* for trend < 0.01) increased significantly. However, consumption of less fat decreased (*p* for trend = 0.01). Other methods did not change significantly between 1999–2000 and 2017–2020 (Figure 5B, Table S8).

Eating less sugar (OR, 2.16; 95% CI, 1.33 to 3.53; *p* < 0.01) and taking prescription medications (OR, 2.55; 95% CI, 1.00 to 6.47; *p* < 0.05) were associated with achieving ≥ 5% weight loss, while using diet foods was associated with unsuccessful weight loss (OR, 0.44; 95% CI, 0.25–0.78; *p* = 0.01). Achieving ≥ 10% weight loss was more likely among those taking prescribed medications (OR, 3.75; 95% CI, 1.37–10.31; *p* = 0.01) (Tables S9 and S10).

### 3.5 Sensitivity Analysis

Using an alternative success metric of a ≥ 0.2 reduction in BMI *z*‐score, 34.34% achieved this threshold overall. Success was more common in boys than girls (40.28% vs. 29.30%; *p* < 0.01). Success rates differed by race/ethnicity (*p* < 0.01) and by weight status (*p* < 0.01), with adolescents with overweight achieving the threshold more often than those with obesity (36.81% vs. 32.66%; Table S11). Across NHANES cycles from 1999 through 2023, there was no significant temporal trend in achieving ≥ 0.2 BMI *z*‐score reduction among adolescents attempting weight loss (adjusted *p* for trend = 0.38; Table S12). Similarly, trend tests were not statistically significant within sex and weight‐status strata (Table S12).

In multivariable logistic regression analyses evaluating weight loss measures and BMI *z*‐score‐defined success, decreasing sugar intake was associated with achieving ≥ 0.2 BMI *z*‐score reduction (adjusted OR, 1.89; 95% CI, 1.16 to 3.08; *p* = 0.01), and taking prescribed diet pills was also associated with success in the full model (adjusted OR, 2.73; 95% CI, 1.02 to 7.32; *p* < 0.05; Table S13). Exercise was not associated with BMI *z*‐score‐defined success (adjusted OR, 0.99; 95% CI, 0.68 to 1.44; *p* = 0.94; Table S13).

## 4 Discussion

This study provided a comprehensive analysis of prevalence and trends in weight loss attempts, clinically significant weight loss achievement, and commonly used weight loss strategies among US adolescents with overweight or obesity. We found that the prevalence of weight loss attempts increased significantly from 53.88% during 1999–2000 to 65.7% during 2021–2023. Despite this, the proportion achieving clinically significant weight loss remained low. The most frequently reported weight loss strategies included exercising, eating less food, drinking water, and reducing junk food consumption. We also observed that reducing sugar intake and taking prescription medications were associated with achieving significant weight loss.

Our results demonstrated that girls consistently reported higher rates of weight loss attempts compared to boys, although the increase over time was more pronounced among boys. Similar patterns were observed in studies from New Zealand and the UK [24, 25]. Changing media representations of male body image standards in recent years may have contributed to greater weight loss efforts among boys [26, 27]. We also observed racial disparities in weight loss attempts, with Mexican Americans adolescents and other racial groups reporting higher prevalence than White and Black adolescents. These differences may reflect variation in cultural factors and societal norms that shape weight control behaviors [28, 29]. In addition, disparities in health care access and potential provider bias may contribute to differences in weight loss behaviors and outcomes across groups, including differences in counseling, referral patterns, and utilization of evidence‐based treatments [30].

Despite the increase in weight loss attempts, the prevalence of achieving clinically significant weight loss remained low over time. Only 12.58% achieved a ≥ 10% weight loss, and participants with overweight were more likely to achieve this target than those with obesity. Adolescents with obesity may face greater barriers to sustained weight loss, such as stigma, depression, and low self‐esteem [31], which could undermine adherence to lifestyle change. Among boys with overweight or obesity, the prevalence of achieving both ≥ 5% and ≥ 10% weight losses increased, while these rates declined among girls. Boys may have physiological advantages to lose weight compared with girls, including greater accrual of lean mass during late adolescence and hormonal differences that may promote muscle gain and fat loss [32].

When using an alternative success metric of a ≥ 0.2 reduction in BMI *z*‐score among adolescents attempting weight loss, 34.34% achieved this threshold, with higher success among boys than girls and significant differences by race/ethnicity and weight status. However, consistent with our primary findings, BMI *z*‐score‐defined success did not show significant improvement over time, and trends were similarly nonsignificant within sex and weight‐status strata. Growth and puberty can affect how weight change is interpreted in adolescents. During this period, increases in height and lean mass may occur, so body weight may stay the same or even increase even if body fat decreases [33]. As a result, achieving a ≥ 5% or a ≥ 10% reduction in body weight may be more difficult for some adolescents, even when their overall body composition is improving.

Evidence showed that exercise can effectively promote weight loss [34]. In our study, exercise remained the most common weight loss strategy among both boys and girls. However, self‐reported exercise was not significantly associated with clinically significant weight loss. This may reflect limitations of NHANES, which did not capture key exercise parameters (mode, frequency, intensity, duration, volume, progression, or adherence). Adolescents who reported “exercising” may have engaged in activity that was insufficient in intensity or duration to produce clinically meaningful changes.

Additionally, we observed that UWCBs remained prevalent, potentially because they were easy to initiate or may yield rapid short‐term results [35]. However, these behaviors have been unsustainable and have been linked to adverse outcomes, including eating disorders and long‐term weight‐related issues [36, 37]. In our multivariable analyses, UWCBs were not associated with successful weight loss, potentially due to their poor long‐term sustainability. Medications have demonstrated the most significant effects on BMI reduction [38]. Consistent with this evidence, our study identified a strong positive association between the use of prescription medications and achieving significant weight loss. Despite this, only 1.78% of participants in our cohort reported using prescription medications for weight loss. Additionally, 6.8% of participants reported using nonprescription products, consistent with previous studies showing a prevalence of 5.5% among adolescents [39]. However, nonprescription products have been associated with adverse outcomes [39]. Bariatric surgery, while highly effective for achieving sustained weight loss and improving the quality of life in adolescents with severe obesity, remains significantly underutilized [40]. No participants in our study reported undergoing weight loss surgery, consistent with prior data indicating that the rate of bariatric procedures among adolescents was low [41].

These patterns demonstrated a critical gap in the use of evidence‐based weight management in this population. One contributing factor may be the stigma associated with seeking professional treatment, leading many adolescents to use unregulated or easily accessible products [42]. To address this gap, comprehensive, multicomponent behavioral interventions, including dietary modifications, physical activity, and behavioral counseling, may effectively promote weight loss in adolescents [43]. Normalizing conversations about weight management and offering personalized, evidence‐informed solutions may help improve outcomes and reduce reliance on ineffective or potentially harmful alternatives [44]. Increased engagement by physicians and other health care providers is important in guiding adolescents toward safe, evidence‐based weight loss strategies.

This study has several strengths. First, it utilized nationally representative data, ensuring that the findings are generalizable to the US adolescent population. Second, the extended study period from 1999 to 2023 allowed for robust trend analyses, providing valuable insights into changes in weight loss attempts and strategies over time. Third, the study comprehensively evaluated both HWCBs and UWCBs, offering a nuanced perspective on the strategies employed by adolescents. Fourth, it identified associations between specific weight loss strategies and successful weight loss, which can inform evidence‐based interventions. Fifth, we conducted sensitivity analyses using an alternative success metric (≥ 0.2 reduction in BMI *z*‐score) to account for adolescent growth and maturation and to assess the robustness of findings across different definitions of weight loss success. However, this study has some limitations that must be considered when interpreting the results. First, self‐reported data on weight, weight loss attempts, and strategies may be subject to recall bias. The lack of information on the intensity, frequency, and adherence to reported weight loss strategies may obscure their true association with achieving clinically significant weight loss. Longitudinal studies are needed to examine the long‐term sustainability and effectiveness of different weight loss strategies, particularly in achieving and maintaining clinically significant weight loss.

## 5 Conclusion

The prevalence of weight loss attempts increased significantly over the past two decades, yet achievement of clinically significant weight loss remained low. Exercise and healthy dietary modifications were the most reported strategies. The low utilization of prescription medications and bariatric surgery demonstrated a gap in addressing adolescent obesity. Evidence‐based approaches are needed to promote sustainable and equitable weight management among adolescents with overweight or obesity.

## Author Contributions

Study concept and design: Ligang Liu, Guang Xiong, Min Liang, Milap C. Nahata. Acquisition of data: Ligang Liu, Guang Xiong. Analysis and interpretation of data: Ligang Liu, Guang Xiong, Hao Zhao, Hekai Shi. Drafting of the manuscript: Ligang Liu, Guang Xiong. Critical revision of the manuscript for important intellectual content: Hao Zhao, Hekai Shi, Min Liang, Milap C. Nahata. Study supervision: Min Liang, Milap C. Nahata.

## Conflicts of Interest

The authors declare no conflicts of interest.

## Supporting information


**Table S1:** Characteristics of Participants by Survey Year.
**Table S2:** Percentage of participants trying to lose weight, stratified by gender, race, and BMI.
**Table S3:** Percentage of participants achieving ≥ 5% weight loss among those attempting to lose weight, stratified by sex, race, and BMI.
**Table S4:** Trends in achieving ≥ 5% weight loss among adolescents (16–19 years) attempting weight loss, 1999‐August 2023: overall and by sex and weight status.
**Table S5:** Percentage of participants achieving ≥ 10% weight loss among those attempting to lose weight, stratified by sex, race, and BMI.
**Table S6:** Trends in achieving ≥ 10% weight loss among adolescents (16–19 years) attempting weight loss, 1999‐August 2023: overall and by sex and weight status.
**Table S7:** Prevalence of weight loss measures among adolescents attempting to lose weight.
**Table S8:** Trend of weight loss measures among adolescents attempting to lose weight by year.
**Table S9:** Backward elimination multivariate logistic regression analysis of the association between weight loss measures and successful weight loss.
**Table S10:** Multivariate logistic regression analysis of the association between weight loss measures and successful weight loss.
**Table S11:** Percentage of participants achieving ≥ 0.2 reduction in BMI *z*‐score among those attempting to lose weight, stratified by sex, race, and BMI.
**Table S12:** Trends in ≥ 0.2 BMI *z*‐score reduction among adolescents (16–19 years) attempting weight loss, 1999‐August 2023: overall and by sex and weight status.
**Table S13:** Multivariate logistic regression analysis of the association between weight‐loss measures and successful weight loss (≥ 0.2 BMI *z*‐score reduction).

## Data Availability

The NHANES data in this study are sourced from the Centers for Disease Control and Prevention, and all data are freely accessible at: https://wwwn.cdc.gov/nchs/nhanes/.
