# Supplementary material for: Trends in Weight Loss Attempts and Strategies Among US Adolescents With Overweight or Obesity
Source: Obesity (Silver Spring). 2026 Apr 5;34(5):1101–14. doi: 10.1002/oby.70184 (PMC13116063; doi:10.1002/oby.70184)
Supplement: Supplementary file 1 — Table S1: Characteristics of Participants by Survey Year. Table S2: Percentage of participants trying to lose weight, stratified by gender, race, and BMI. Table S3: Percentage of participants achieving ≥ 5% weight loss among those attempting to lose weight, stratified by sex, race, and BMI. Table S4: Trends in achieving ≥ 5% weight loss among adolescents (16–19 years) attempting weight loss, 1999‐August 2023: overall and by sex and weight status. Table S5: Percentage of participants achieving ≥ 10% weight loss among those attempting to lose weight, stratified by sex, race, and BMI. Table S6: Trends in achieving ≥ 10% weight loss among adolescents (16–19 years) attempting weight loss, 1999‐August 2023: overall and by sex and weight status. Table S7: Prevalence of weight loss measures among adolescents attempting to lose weight. Table S8: Trend of weight loss measures among adolescents attempting to lose weight by year. Table S9: Backward elimination multivariate logistic regression analysis of the association between weight loss measures and successful weight loss. Table S10: Multivariate logistic regression analysis of the association between weight loss measures and successful weight loss. Table S11: Percentage of participants achieving ≥ 0.2 reduction in BMI z‐score among those attempting to lose weight, stratified by sex, race, and BMI. Table S12: Trends in ≥ 0.2 BMI z‐score reduction among adolescents (16–19 years) attempting weight loss, 1999‐August 2023: overall and by sex and weight status. Table S13: Multivariate logistic regression analysis of the association between weight‐loss measures and successful weight loss (≥ 0.2 BMI z‐score reduction). [file OBY-34-1101-s001.docx]

**Table S1. Characteristics of Participants by Survey Year**

| **Characteristics** | **NHANES participants, N (weighted %)** | | | | | | | | | | | | **P-value** |
| --- | --- | --- | --- | --- | --- | --- | --- | --- | --- | --- | --- | --- | --- |
|  | **Total (N=2708)** | **1999-2000 (N=302)** | **2001-2002 (N=312)** | **2003-2004 (N=335)** | **2005-2006 (N=360)** | **2007-2008 (N=167)** | **2009-2010 (N=198)** | **2011-2012 (N=163)** | **2013-2014 (N=217)** | **2015-2016 (N=192)** | **2017-March 2020 (N=293)** | **August 2021-August 2023 (N=169)** |  |
| Age (years) | 17.37 ± 0.03 | 17.47 ± 0.09 | 17.29 ± 0.06 | 17.45 ± 0.09 | 17.46 ± 0.11 | 17.27 ± 0.11 | 17.35 ± 0.08 | 17.42 ± 0.15 | 17.43 ± 0.08 | 17.23 ± 0.06 | 17.38 ± 0.09 | 17.34 ± 0.11 | 0.42 |
| Sex |  |  |  |  |  |  |  |  |  |  |  |  | 0.32 |
| Boy | 1381 (52.56) | 159 (55.19) | 168 (57.02) | 181 (57.61) | 174 (52.85) | 93 (57.73) | 107 (55.16) | 85 (51.44) | 110 (55.18) | 83 (42.11) | 140 (48.05) | 81 (51.01) |  |
| Girl | 1327 (47.44) | 143 (44.81) | 144 (42.98) | 154 (42.39) | 186 (47.15) | 74 (42.27) | 91 (44.84) | 78 (48.56) | 107 (44.82) | 109 (57.89) | 153 (51.95) | 88 (48.99) |  |
| Race |  |  |  |  |  |  |  |  |  |  |  |  | 0.06 |
| Non-Hispanic White | 709 (53.91) | 50 (48.98) | 86 (60.02) | 93 (67.00) | 82 (59.95) | 51 (61.26) | 51 (50.52) | 35 (48.79) | 55 (52.37) | 50 (50.21) | 93 (50.60) | 63 (45.67) |  |
| Non-Hispanic Black | 812 (15.92) | 81 (15.27) | 90 (13.66) | 125 (15.85) | 142 (19.88) | 49 (17.38) | 57 (20.28) | 65 (20.10) | 54 (14.68) | 40 (12.87) | 81 (14.27) | 28 (12.96) |  |
| Mexican American | 784 (15.45) | 138 (14.99) | 104 (9.53) | 105 (11.26) | 113 (11.34) | 37 (11.24) | 57 (17.44) | 26 (13.21) | 55 (16.47) | 57 (20.16) | 62 (23.06) | 30 (14.74) |  |
| Others | 403 (14.72) | 33 (20.76) | 32 (16.79) | 12 (5.90) | 23 (8.83) | 30 (10.12) | 33 (11.77) | 37 (17.89) | 53 (16.48) | 45 (16.76) | 57 (12.06) | 48 (26.63) |  |
| Family income |  |  |  |  |  |  |  |  |  |  |  |  | 0.26 |
| Low Income | 1230 (35.25) | 155 (48.43) | 142 (37.69) | 154 (37.37) | 152 (27.64) | 68 (31.64) | 93 (38.03) | 81 (37.78) | 111 (39.31) | 84 (32.26) | 130 (33.10) | 60 (29.68) |  |
| Middle Income | 997 (38.37) | 105 (34.25) | 112 (33.62) | 127 (38.31) | 144 (43.63) | 60 (33.79) | 74 (34.78) | 56 (42.55) | 73 (40.98) | 75 (40.40) | 97 (34.23) | 74 (45.96) |  |
| High Income | 481 (26.37) | 42 (17.33) | 58 (28.69) | 54 (24.31) | 64 (28.73) | 39 (34.57) | 31 (27.19) | 26 (19.66) | 33 (19.71) | 33 (27.34) | 66 (32.68) | 35 (24.36) |  |
| BMI (kg/m^2^) | 31.63 ± 0.16 | 30.76 ± 0.52 | 30.63 ± 0.36 | 31.55 ± 0.60 | 31.23 ± 0.55 | 31.44 ± 0.62 | 31.52 ± 0.53 | 32.12 ± 0.75 | 32.89 ± 0.49 | 31.24 ± 0.58 | 31.98 ± 0.37 | 32.02 ± 0.50 | 0.04 |
| Initial weight (pounds) | 188.74 ± 1.09 | 186.97 ± 2.83 | 185.68 ± 1.85 | 190.79 ± 2.61 | 185.84 ± 3.58 | 191.04 ± 4.11 | 191.60 ± 4.04 | 192.27 ± 5.04 | 192.21 ± 5.09 | 179.69 ± 2.79 | 188.77 ± 2.67 | 191.53 ± 3.53 | 0.12 |
| Current Weight (pounds) | 194.27 ± 1.13 | 192.00 ± 3.30 | 191.50 ± 2.28 | 196.67 ± 3.49 | 192.47 ± 4.25 | 193.90 ± 3.03 | 196.03 ± 4.32 | 197.19 ± 5.20 | 201.06 ± 4.90 | 184.62 ± 3.00 | 195.56 ± 2.74 | 194.79 ± 3.56 | 0.16 |
| Weight change (pounds) | -5.54 ± 0.53 | -5.03 ± 1.72 | -5.82 ± 1.98 | -5.88 ± 1.61 | -6.63 ± 2.10 | -2.85 ± 2.19 | -4.43 ± 1.77 | -4.93 ± 1.73 | -8.85 ± 1.45 | -4.93 ± 1.29 | -6.79 ± 1.41 | -3.27 ± 1.89 | 0.44 |
| Weight Status |  |  |  |  |  |  |  |  |  |  |  |  | 0.92 |
| Overweight | 1229 (46.15) | 142 (52.20) | 147 (47.68) | 154 (48.02) | 158 (47.19) | 74 (42.99) | 90 (43.50) | 82 (48.00) | 99 (42.74) | 98 (51.66) | 117 (44.02) | 68 (42.48) |  |
| Obesity | 1479 (53.85) | 160 (47.80) | 165 (52.32) | 181 (51.98) | 202 (52.81) | 93 (57.01) | 108 (56.50) | 81 (52.00) | 118 (57.26) | 94 (48.34) | 176 (55.98) | 101 (57.52) |  |
| Tried to lose weight |  |  |  |  |  |  |  |  |  |  |  |  | < 0.01 |
| No | 1068 (38.72) | 141 (46.12) | 135 (43.01) | 149 (39.14) | 153 (40.56) | 62 (44.08) | 96 (56.42) | 66 (42.27) | 68 (32.24) | 53 (27.42) | 89 (31.21) | 56 (34.30) |  |
| Yes | 1640 (61.28) | 161 (53.88) | 177 (56.99) | 186 (60.86) | 207 (59.44) | 105 (55.92) | 102 (43.58) | 97 (57.73) | 149 (67.76) | 139 (72.58) | 204 (68.79) | 113 (65.70) |  |

Continuous variables are expressed as the weighted mean ± standard error; categorical variables are expressed as unweighted numbers (weighted percentages).

Abbreviations: NHANES, National Health and Nutrition Examination Survey; BMI, body mass index.

**Table S2. Percentage of Participants Trying to Lose Weight, Stratified by Gender, Race, and BMI**

| **Stratification** | **Participants, N (weighted %) (95% CI)** | | | **P-value** |
| --- | --- | --- | --- | --- |
|  | **Total (N=2708)** | **Boys (N=1381)** | **Girls (N=1327)** |  |
| Overall | 1640 (61.28) (56.43-66.13) | 714 (53.50) (49.68-57.32) | 926 (69.91) (66.45-73.37) | < 0.01 |
| By race |  |  |  | < 0.01 |
| Non-Hispanic White | 410 (57.60) (53.68-61.53) | 191 (49.00) (43.43-54.58) | 219 (67.77) (61.75-73.79) |  |
| Non-Hispanic Black | 460 (59.72) (55.81-63.62) | 159 (45.75) (40.02-51.47) | 301 (70.89) (66.17-75.61) |  |
| Mexican American | 495 (69.53) (64.77-74.30) | 230 (65.15) (57.91-72.39) | 265 (74.55) (68.73-80.38) |  |
| Others | 275 (67.78) (60.96-74.61) | 134 (64.68) (55.32-74.04) | 141 (71.52) (61.59-81.44) |  |
| By BMI |  |  |  | < 0.01 |
| Obesity | 976 (67.69) (64.69-70.70) | 480 (63.05) (58.32-67.77) | 496 (73.59) (69.09-78.10) |  |
| Overweight | 664 (53.80) (50.38-57.21) | 234 (40.68) (35.78-45.58) | 430 (66.22) (61.46-70.97) |  |

Abbreviations: CI, Confidence interval; BMI, body mass index.

**Table S3. Percentage of Participants Achieving ≥5% Weight Loss Among Those Attempting to Lose Weight, Stratified by Sex, Race, and BMI**

| **Stratification** | **Participants, N (weighted %) (95% CI)** | | | **P-value** |
| --- | --- | --- | --- | --- |
|  | **Total (N=1640)** | **Boys (N=714)** | **Girls (N=** **926)** |  |
| Overall | 409 (26.96) (23.33-30.60) | 204 (29.07) (24.41-33.73) | 205 (25.18) (21.14-29.21) | 0.21 |
| By race |  |  |  | 0.05 |
| Non-Hispanic White | 127 (30.33) (25.11-35.54) | 61 (29.96) (22.65-37.28) | 66 (30.64) (23.61-37.67) |  |
| Non-Hispanic Black | 107 (23.27) (19.43-27.10) | 50 (30.97) (23.36-38.57) | 57 (19.30) (14.53-24.07) |  |
| Mexican American | 105 (21.00) (15.84-26.16) | 55 (24.68) (16.83-32.54) | 50 (17.31) (10.21-24.40) |  |
| Others | 70 (26.44) (21.00-31.89) | 38 (29.96) (21.75-38.17) | 32 (22.61) (15.29-29.94) |  |
| By BMI |  |  |  | 0.01 |
| Obesity | 216 (23.51) (19.69-27.33) | 125 (26.31) (20.64-31.99) | 91 (20.46) (15.55-25.38) |  |
| Overweight | 193 (32.03) (26.90-37.17) | 79 (34.80) (27.28-42.33) | 114 (30.42) (24.16-36.68) |  |

Abbreviations: CI, Confidence interval; BMI, body mass index.

**Table S4.** **Trends in Achieving ≥5% Weight Loss Among Adolescents (16-19 Years) Attempting Weight Loss, 1999-August 2023: Overall and by Sex and Weight Status**

| **Weight loss success rates** | **1999-2000** | **2001-2002** | **2003-2004** | **2005-2006** | **2007-2008** | **2009-2010** | **2011-2012** | **2013-2014** | **2015-2016** | **2017-March 2020** | **August 2021-August 2023** | **P for trend *** | **Average annual percent change (95% confidence interval)** |
| --- | --- | --- | --- | --- | --- | --- | --- | --- | --- | --- | --- | --- | --- |
| **All participants** |  |  |  |  |  |  |  |  |  |  |  |  |  |
| Unadjusted | 28.38 ± 5.77 | 29.07 ± 4.58 | 27.94 ± 5.31 | 27.22 ± 4.86 | 26.45 ± 6.45 | 40.00 ± 7.02 | 29.63 ± 5.65 | 16.01 ± 3.00 | 24.19 ± 3.74 | 22.34 ± 4.25 | 36.80 ± 5.44 | 0.79 | 0.01 (-2.33 to 2.48) |
| Adjusted | 27.30 ± 5.87 | 30.86 ± 5.28 | 28.23 ± 4.88 | 27.37 ± 5.22 | 28.94 ± 5.51 | 40.40 ± 5.98 | 29.96 ± 6.11 | 16.51 ± 3.51 | 25.98 ± 3.62 | 23.32 ± 4.15 | 35.80 ± 4.87 | 1.00 | 0.05 (-1.93 to 2.25) |
| **Overweight** |  |  |  |  |  |  |  |  |  |  |  |  |  |
| Unadjusted | 33.02 ± 10.91 | 32.24 ± 6.67 | 29.19 ± 8.19 | 41.15 ± 8.99 | 15.98 ± 7.54 | 38.75 ± 8.66 | 37.38 ± 12.07 | 16.78 ± 3.38 | 37.21 ± 7.68 | 29.59 ± 7.14 | 40.06 ± 8.42 | 0.78 | 0.04 (-3.16 to 3.63) |
| Adjusted | 32.29 ± 10.93 | 34.45 ± 7.58 | 29.83 ± 7.03 | 41.57 ± 9.44 | 19.63 ± 7.89 | 39.19 ± 8.33 | 35.33 ± 9.86 | 18.09 ± 3.93 | 39.38 ± 8.81 | 32.06 ± 7.20 | 39.99 ± 7.93 | 0.55 | 0.25 (-2.45 to 3.23) |
| **Obesity** |  |  |  |  |  |  |  |  |  |  |  |  |  |
| Unadjusted | 24.80 ± 6.86 | 25.17 ± 4.69 | 26.99 ± 6.88 | 15.24 ± 3.03 | 33.00 ± 7.99 | 40.54 ± 9.35 | 24.23 ± 8.05 | 15.63 ± 4.22 | 13.86 ± 5.64 | 18.26 ± 4.71 | 34.75 ± 5.76 | 0.66 | 0.84 (-3.62 to 5.04) |
| Adjusted | 23.45 ± 5.08 | 28.55 ± 5.84 | 26.69 ± 6.43 | 15.03 ± 2.58 | 34.31 ± 6.85 | 42.00 ± 7.16 | 25.07 ± 7.79 | 16.14 ± 4.14 | 16.29 ± 5.48 | 19.97 ± 4.88 | 34.06 ± 6.26 | 0.68 | 0.61 (-4.78 to 5.31) |
| **Boys** |  |  |  |  |  |  |  |  |  |  |  |  |  |
| Unadjusted | 28.34 ± 6.44 | 33.18 ± 7.18 | 31.66 ± 7.68 | 19.57 ± 3.87 | 39.06 ± 8.34 | 52.91 ± 10.35 | 37.82 ± 10.03 | 9.50 ± 3.24 | 25.44 ± 5.71 | 26.49 ± 8.03 | 35.81 ± 7.63 | 0.67 | 0.07 (-3.70 to 3.69) |
| Adjusted | 25.13 ± 5.86 | 35.39 ± 6.47 | 33.58 ± 6.92 | 20.56 ± 4.61 | 39.69 ± 9.13 | 50.74 ± 8.69 | 38.08 ± 8.62 | 7.80 ± 2.93 | 25.29 ± 6.59 | 28.52 ± 7.02 | 36.37 ± 7.67 | 0.72 | 0.07 (-3.71 to 3.60) |
| **Girls** |  |  |  |  |  |  |  |  |  |  |  |  |  |
| Unadjusted | 28.41 ± 7.72 | 25.89 ± 5.04 | 23.44 ± 5.83 | 32.19 ± 6.41 | 17.09 ± 6.93 | 29.33 ± 7.72 | 22.57 ± 8.38 | 24.34 ± 6.11 | 23.44 ± 5.88 | 18.96 ± 4.27 | 37.91 ± 9.89 | 0.98 | -0.19 (-2.77 to 2.21) |
| Adjusted | 28.17 ± 8.08 | 27.22 ± 5.53 | 21.91 ± 5.57 | 32.81 ± 6.22 | 16.75 ± 4.23 | 28.64 ± 7.55 | 19.04 ± 6.47 | 24.04 ± 6.75 | 26.47 ± 5.96 | 19.20 ± 4.06 | 30.32 ± 6.37 | 0.81 | -0.32 (-3.63 to 2.93) |
| **Boys Overweight** |  |  |  |  |  |  |  |  |  |  |  |  |  |
| Unadjusted | 30.34 ± 11.43 | 28.84 ± 11.81 | 39.46 ± 10.65 | 29.76 ± 11.88 | 35.24 ± 12.80 | 64.66 ± 13.99 | 37.01 ± 13.71 | 21.26 ± 7.18 | 48.19 ± 14.15 | 35.05 ± 12.03 | 29.40 ± 12.72 | 0.92 | 0.14 (-3.41 to 3.95) |
| Adjusted | 18.11 ± 7.84 | 23.86 ± 8.62 | 41.93 ± 7.78 | 29.95 ± 9.49 | 32.28 ± 11.81 | 60.77 ± 12.59 | 39.16 ± 10.34 | 22.03 ± 9.29 | 41.98 ± 8.28 | 38.83 ± 6.05 | 29.08 ± 11.48 | 0.69 | 0.72 (-2.92 to 5.33) |
| **Boys Obesity** |  |  |  |  |  |  |  |  |  |  |  |  |  |
| Unadjusted | 27.17 ± 10.08 | 37.76 ± 7.42 | 25.33 ± 7.49 | 13.87 ± 4.58 | 40.88 ± 11.31 | 50.05 ± 12.44 | 38.13 ± 13.40 | 5.51 ± 2.25 | 13.56 ± 8.88 | 22.71 ± 8.74 | 37.87 ± 8.16 | 0.70 | -0.12 (-7.85 to 6.79) |
| Adjusted | 27.31 ± 7.42 | 47.21 ± 8.60 | 25.15 ± 7.84 | 13.41 ± 4.65 | 41.79 ± 10.33 | 44.55 ± 10.70 | 47.97 ± 10.56 | 4.52 ± 1.63 | 15.24 ± 8.56 | 23.98 ± 8.04 | 37.94 ± 9.36 | 0.68 | -1.08 (-10.90 to 7.82) |
| **Girls Overweight** |  |  |  |  |  |  |  |  |  |  |  |  |  |
| Unadjusted | 34.29 ± 12.74 | 34.57 ± 7.17 | 15.64 ± 6.86 | 46.15 ± 10.43 | 5.29 ± 4.09 | 27.80 ± 8.92 | 37.54 ± 15.62 | 13.43 ± 5.57 | 32.67 ± 8.36 | 26.21 ± 9.00 | 45.34 ± 9.85 | 0.73 | 0.55 (-2.76 to 4.09) |
| Adjusted | 28.75± 12.70 | 36.95 ± 8.11 | 15.30 ± 6.41 | 46.50 ± 10.67 | 7.72 ± 4.11 | 26.72 ± 7.11 | 30.36 ± 10.69 | 15.35 ± 4.96 | 35.96 ± 9.55 | 25.11 ± 7.64 | 41.00 ± 8.74 | 0.49 | 0.07 (-4.17 to 4.61) |
| **Girls Obesity** |  |  |  |  |  |  |  |  |  |  |  |  |  |
| Unadjusted | 23.07 ± 8.29 | 13.88 ± 3.83 | 28.86 ± 10.13 | 16.44 ± 4.92 | 26.04 ± 8.64 | 30.28 ± 11.07 | 5.91 ± 3.70 | 32.69 ± 7.48 | 14.11 ± 6.26 | 14.05 ± 4.65 | 28.95 ± 15.27 | 0.72 | 0.69 (-6.73 to 8.17) |
| Adjusted | 21.10 ± 6.27 | 16.78 ± 4.60 | 24.52 ± 7.68 | 17.05 ± 4.28 | 24.59 ± 5.28 | 29.51 ± 8.35 | 5.64 ± 3.07 | 31.42 ± 8.14 | 17.70 ± 4.93 | 15.46 ± 4.74 | 18.82 ± 9.24 | 0.93 | -0.26 (-4.95 to 4.08) |

Participation rates are presented as weighted percentages ± standard error. Adjusted rates are age-standardized using the 2000 U.S. Census population for adolescents aged 16-19 years. In the adjusted model, P for trend was additionally adjusted for age, sex, race/ethnicity, and family income. The average annual percent change is expressed as "estimate (95% confidence interval)" to represent the average yearly change over the study period.

**Table S5. Percentage of Participants Achieving ≥10% Weight Loss Among Those Attempting to Lose Weight, Stratified by Sex, Race, and BMI**

| **Stratification** | **Participants, N (weighted percentages) (95% CI)** | | | **P-value** |
| --- | --- | --- | --- | --- |
|  | **Total (N=1640)** | **Boys (N=714)** | **Girls (N=** **926)** |  |
| Overall | 191 (12.58) (9.97-15.19) | 100 (14.47) (10.81-18.13) | 91 (10.98) (7.99-13.96) | 0.14 |
| By race |  |  |  | 0.31 |
| Non-Hispanic White | 59 (14.06) (10.07-18.05) | 30 (15.19) (9.34-21.04) | 29 (13.09) (7.81-18.38) |  |
| Non-Hispanic Black | 54 (11.01) (8.04-13.99) | 25 (13.04) (7.52-18.56) | 29 (9.96) (6.39-13.54) |  |
| Mexican American | 45 (9.08) (5.45-12.71) | 26 (12.34) (6.11-18.56) | 19 (5.82) (2.45- 9.19) |  |
| Others | 33 (13.23) (8.38-18.08) | 19 (15.58) (7.70-23.47) | 14 (10.68) (5.38-15.97) |  |
| By BMI |  |  |  | 0.02 |
| Obesity | 98 (10.28) (7.55-13.00) | 57 (11.92) (7.82-16.02) | 41 (8.48) (5.08-11.89) |  |
| Overweight | 93 (15.96) (11.80-20.13) | 43 (19.77) (13.21-26.33) | 50 (13.75) (8.54-18.96) |  |

Abbreviations: CI, Confidence interval; BMI, body mass index.

**Table S6.** **Trends in Achieving ≥10% Weight Loss** **Among Adolescents (16-19 Years) Attempting Weight Loss, 1999-August 2023: Overall and by Sex and Weight Status**

| **Weight loss success rates** | **1999-2000** | **2001-2002** | **2003-2004** | **2005-2006** | **2007-2008** | **2009-2010** | **2011-2012** | **2013-2014** | **2015-2016** | **2017-March 2020** | **August 2021-August 2023** | **P for trend *** | **Average annual percent change (95% confidence interval)** |
| --- | --- | --- | --- | --- | --- | --- | --- | --- | --- | --- | --- | --- | --- |
| **All participants** |  |  |  |  |  |  |  |  |  |  |  |  |  |
| Unadjusted | 14.77 ± 4.81 | 14.23 ± 3.85 | 14.49 ± 2.36 | 14.08 ± 4.01 | 12.32 ± 4.70 | 20.11 ± 4.84 | 12.65 ± 4.84 | 4.98 ± 2.12 | 6.98 ± 3.29 | 14.12 ± 3.65 | 14.65 ± 4.88 | 0.50 | -0.86 (-4.08 to 1.71) |
| Adjusted | 13.83 ± 4.89 | 16.03 ± 4.49 | 15.08 ± 2.58 | 14.23 ± 4.05 | 15.42 ± 4.39 | 21.86 ± 4.66 | 12.63 ± 4.71 | 4.42 ± 1.73 | 7.89 ± 4.05 | 15.30 ± 3.63 | 16.95 ± 5.38 | 0.61 | -0.51 (-4.44 to 2.97) |
| **Overweight** |  |  |  |  |  |  |  |  |  |  |  |  |  |
| Unadjusted | 24.40 ± 10.98 | 13.97 ± 5.86 | 15.61 ± 4.20 | 20.94 ± 6.91 | 10.01 ± 6.52 | 27.58 ± 9.81 | 24.93 ± 11.64 | 5.50 ± 2.97 | 5.40 ± 2.35 | 18.72 ± 7.16 | 18.41 ± 5.12 | 0.59 | -0.80 (-5.90 to 4.46) |
| Adjusted | 22.77 ± 10.47 | 15.85 ± 6.83 | 16.65 ± 5.17 | 20.76 ± 6.97 | 14.01 ± 6.37 | 28.64 ± 8.96 | 21.09 ± 8.23 | 5.46 ± 2.97 | 5.34 ± 1.82 | 22.34 ± 7.24 | 23.82 ± 6.68 | 0.68 | -0.29 (-5.01 to 5.16) |
| **Obesity** |  |  |  |  |  |  |  |  |  |  |  |  |  |
| Unadjusted | 7.35 ± 3.58 | 14.54 ± 3.31 | 13.64 ± 3.43 | 8.17 ± 2.91 | 13.77 ± 6.02 | 16.95 ± 6.19 | 4.08 ± 2.32 | 4.72 ± 2.86 | 8.23 ± 4.51 | 11.53 ± 4.56 | 12.27 ± 5.25 | 0.81 | -1.20 (-6.95 to 2.65) |
| Adjusted | 6.85 ± 3.04 | 16.94 ± 4.37 | 14.53 ± 4.20 | 8.32 ± 2.72 | 15.33 ± 5.17 | 21.06 ± 6.44 | 4.15 ± 2.02 | 4.40 ± 2.08 | 10.02 ± 5.23 | 13.04 ± 4.81 | 14.49 ± 5.96 | 0.84 | -0.73 (-5.86 to 3.59) |
| **Boys** |  |  |  |  |  |  |  |  |  |  |  |  |  |
| Unadjusted | 16.67 ± 3.65 | 17.45 ± 6.19 | 17.78 ± 3.01 | 10.19 ± 4.85 | 18.44 ± 6.49 | 25.91 ± 7.56 | 5.89 ± 2.66 | 4.42 ± 2.16 | 8.82 ± 5.37 | 19.46 ± 7.28 | 18.04 ± 8.02 | 0.91 | -1.16 (-7.13 to 2.43) |
| Adjusted | 15.42 ± 4.24 | 21.11 ± 5.73 | 18.55 ± 2.69 | 10.81 ± 4.30 | 14.73 ± 5.21 | 25.98 ± 6.86 | 5.13 ± 1.86 | 4.31 ± 2.44 | 9.85 ± 6.60 | 22.06 ± 5.99 | 19.37 ± 6.69 | 0.96 | -0.42 (-6.69 to 3.93) |
| **Girls** |  |  |  |  |  |  |  |  |  |  |  |  |  |
| Unadjusted | 13.62 ± 7.49 | 11.73 ± 4.26 | 10.51 ± 4.33 | 16.60 ± 4.69 | 7.78 ± 6.10 | 15.33 ± 6.37 | 18.48 ± 8.91 | 5.70 ± 4.09 | 5.86 ± 2.70 | 9.76 ± 3.52 | 10.87 ± 4.02 | 0.30 | -1.79 (-5.72 to 1.90) |
| Adjusted | 11.96 ± 7.22 | 12.74 ± 4.74 | 10.73 ± 4.79 | 17.08 ± 4.05 | 10.50 ± 3.03 | 16.94 ± 6.19 | 16.17 ± 6.87 | 4.72 ± 2.91 | 6.69 ± 3.15 | 10.04 ± 3.33 | 12.08 ± 3.57 | 0.41 | -1.48 (-7.51 to 3.76) |
| **Boys Overweight** |  |  |  |  |  |  |  |  |  |  |  |  |  |
| Unadjusted | 18.83 ± 14.44 | 18.19 ± 10.57 | 21.81 ± 4.97 | 19.39 ± 10.54 | 23.15 ± 12.12 | 51.12 ± 17.48 | 5.77 ± 3.59 | 9.12 ± 5.31 | 7.17 ± 6.66 | 28.34 ± 13.58 | 26.47 ± 12.57 | 0.85 | 0.22 (-5.92 to 5.58) |
| Adjusted | 12.23 ± 8.00 | 14.77 ± 7.57 | 21.52 ± 6.04 | 19.88 ± 6.30 | 21.51 ± 10.19 | 43.77 ± 12.10 | 6.65 ± 4.70 | 11.37 ± 7.41 | 2.62 ± 2.40 | 34.16 ± 7.38 | 22.16 ± 8.46 | 0.53 | 2.07 (-2.70 to 8.14) |
| **Boys Obesity** |  |  |  |  |  |  |  |  |  |  |  |  |  |
| Unadjusted | 15.41 ± 7.09 | 16.67 ± 7.76 | 14.51 ± 4.71 | 5.05 ± 2.35 | 16.21 ± 9.32 | 19.79 ± 8.03 | 5.94 ± 3.87 | 2.83 ± 1.75 | 9.68 ± 6.86 | 15.53 ± 7.81 | 15.32 ± 7.07 | 0.99 | -0.75 (-6.27 to 4.17) |
| Adjusted | 14.68 ± 6.46 | 25.46 ± 8.77 | 15.10 ± 5.66 | 5.38 ± 2.34 | 10.40 ± 5.51 | 16.87 ± 6.18 | 5.16 ± 2.49 | 2.67 ± 1.57 | 11.84 ± 7.26 | 17.39 ± 6.88 | 17.09 ± 7.31 | 0.96 | -1.07 (-8.49 to 5.88) |
| **Girls Overweight** |  |  |  |  |  |  |  |  |  |  |  |  |  |
| Unadjusted | 27.02 ± 13.47 | 11.08 ± 6.40 | 7.42 ± 4.48 | 21.62 ± 7.74 | 2.72 ± 2.82 | 17.64 ± 8.60 | 33.63 ± 15.85 | 2.81 ± 2.77 | 4.66 ± 1.77 | 12.76 ± 8.11 | 14.42 ± 5.75 | 0.43 | -2.74 (-10.78 to 6.00) |
| Adjusted | 26.46 ± 14.33 | 13.03 ± 7.08 | 7.77 ± 4.45 | 21.32 ± 6.73 | 6.54 ± 3.92 | 18.39 ± 7.15 | 26.42 ± 10.34 | 3.07 ± 2.85 | 5.23 ± 1.88 | 13.91 ± 7.35 | 18.41 ± 6.86 | 0.47 | -1.66 (-9.55 to 6.77) |
| **Girls Obesity** |  |  |  |  |  |  |  |  |  |  |  |  |  |
| Unadjusted | 1.46 ± 0.94 | 12.63 ± 3.95 | 12.65 ± 5.60 | 10.93 ± 4.80 | 11.62 ± 9.25 | 13.89 ± 9.53 | 1.62 ± 1.68 | 7.91 ± 7.16 | 7.06 ± 4.67 | 7.74 ± 3.83 | 6.60 ± 4.38 | 0.52 | -1.19 (-8.48 to 4.58) |
| Adjusted | 1.35 ± 0.82 | 15.89 ± 4.69 | 11.77 ± 4.94 | 10.81 ± 3.51 | 11.76 ± 2.98 | 18.14 ± 6.95 | 2.19 ± 2.51 | 7.18 ± 3.52 | 8.51 ± 5.27 | 8.48 ± 3.81 | 4.28 ± 3.06 | 0.87 | -1.71 (-9.72 to 4.54) |

Participation rates are presented as weighted percentages ± standard error. Adjusted rates are age-standardized using the 2000 U.S. Census population for adolescents aged 16-19 years. In the adjusted model, P for trend was additionally adjusted for age, sex, race/ethnicity, and family income. The average annual percent change is expressed as "estimate (95% confidence interval)" to represent the average yearly change over the study period.

**Table S7. Prevalence of Weight Loss Measures Among Adolescents Attempting to Lose Weight**

| **Weight Loss Measures** | **Participants, N (weighted %) (95% CI)** |
| --- | --- |
| Exercised | 1202 (80.01) (73.24-86.78) |
| Ate less food | 862 (57.09) (50.86-63.32) |
| Drank a lot of water | 709 (52.96) (47.20-58.73) |
| Ate less junk food or fast food | 320 (29.72) (24.84-34.61) |
| Ate more fruits, vegetables, salads | 310 (26.99) (22.40-31.57) |
| Chose low-calorie foods | 328 (24.14) (20.00-28.27) |
| Ate less fat | 384 (23.69) (20.38-27.01) |
| Skipped meals | 378 (22.51) (19.37-25.66) |
| Ate less sugar, candy, sweets | 264 (22.99) (19.08-26.91) |
| Changed eating habits | 263 (21.40) (17.71-25.09) |
| Ate fewer carbohydrates | 136 (11.18) (8.55-13.81) |
| Ate diet products | 168 (11.55) (9.29-13.81) |
| Took non-prescription supplements | 108 (6.80) (4.84-8.75) |
| Followed special diet | 72 (5.81) (3.64-7.98) |
| Used liquid diet formula | 99 (6.15) (4.42-7.88) |
| Joined weight loss program | 41 (3.11) (1.97-4.25) |
| Other method | 30 (1.59) (0.83-2.34) |
| Took prescribed diet pills | 36 (1.78) (0.99-2.57) |
| Used laxatives or vomited | 22 (1.04) (0.52-1.56) |
| Smoke | 7 (0.45) (0.00-0.90) |

Abbreviations: CI, Confidence interval; BMI, body mass index.

**Table S8.** **Trend of Weight Loss Measures Among Adolescents Attempting to Lose Weight by Year**

| **Weight loss measures** | **1999-2000** | **2001-2002** | **2003-2004** | **2005-2006** | **2007-2008** | **2009-2010** | **2011-2012** | **2013-2014** | **2015-2016** | **2017-March 2020** | **P for trend *** | **Average annual percent change (95% confidence interval)** |
| --- | --- | --- | --- | --- | --- | --- | --- | --- | --- | --- | --- | --- |
| **Exercised** |  |  |  |  |  |  |  |  |  |  |  |  |
| unadjusted | 74.70 ± 4.68 | 73.04 ± 5.30 | 78.89 ± 4.04 | 84.93 ± 2.81 | 82.39 ± 5.95 | 79.47 ± 5.41 | 83.59 ± 4.45 | 85.01 ± 4.64 | 79.85 ± 4.52 | 77.56 ± 2.88 | 0.50 | 0.45 (-0.13 to 1.36) |
| adjusted | 75.25 ± 4.56 | 72.34 ± 5.46 | 78.37 ± 3.71 | 84.50 ± 3.15 | 81.08 ± 5.72 | 79.91 ± 4.69 | 83.95 ± 3.78 | 87.21 ± 3.01 | 79.47 ± 4.55 | 76.67 ± 2.96 | 0.53 | 0.01 (-0.43 to 0.67) |
| **Ate less food** |  |  |  |  |  |  |  |  |  |  |  |  |
| unadjusted | 60.58 ± 5.06 | 59.45 ± 5.02 | 64.78 ± 3.52 | 68.60 ± 3.69 | 44.96 ± 6.83 | 59.48 ± 7.15 | 58.26 ± 6.96 | 49.52 ± 7.95 | 54.25 ± 6.87 | 55.53 ± 4.42 | 0.08 | -0.91 (-2.69 to 0.17) |
| adjusted | 59.89 ± 5.47 | 57.95 ± 5.40 | 64.85 ± 3.36 | 69.09 ± 3.73 | 42.83 ± 6.81 | 57.38 ± 6.56 | 56.58 ± 6.24 | 47.66 ± 7.70 | 54.67 ± 6.62 | 55.80 ± 4.28 | 0.12 | -0.96 (-2.86 to 0.23) |
| **Drank a lot of water** |  |  |  |  |  |  |  |  |  |  |  |  |
| unadjusted | 0.70 ± 0.42 | 40.51 ± 8.23 | 48.47 ± 4.29 | 53.17 ± 4.23 | 51.81 ± 8.00 | 48.13 ± 7.93 | 47.65 ± 8.06 | 48.94 ± 3.97 | 65.86 ± 2.98 | 77.87 ± 3.40 | <0.01 | 3.66 (1.72 to 8.01) |
| adjusted | 0.92 ± 0.56 | 38.48 ± 7.40 | 50.39 ± 3.80 | 53.20 ± 4.23 | 52.47 ± 7.32 | 46.83 ± 7.58 | 46.28 ± 7.17 | 50.84 ± 3.46 | 64.34 ± 3.45 | 77.88 ± 3.18 | <0.01 | 3.43 (1.45 to 7.61) |
| **Ate less junk food or fast food** |  |  |  |  |  |  |  |  |  |  |  |  |
| unadjusted | NA | NA | NA | NA | NA | 46.84 ± 6.12 | 45.62 ± 7.11 | 45.31 ± 4.58 | 50.55 ± 5.45 | 60.56 ± 5.05 | 0.03 | 3.53 (-0.81 to 9.42) |
| adjusted | NA | NA | NA | NA | NA | 43.75 ± 4.59 | 44.90 ± 6.58 | 45.07 ± 4.12 | 49.46 ± 5.49 | 60.68 ± 4.74 | 0.08 | 4.10 (0.21 to 8.86) |
| **Ate more fruits, vegetables, salads** |  |  |  |  |  |  |  |  |  |  |  |  |
| unadjusted | NA | NA | NA | 1.36 ± 1.36 | NA | 34.42 ± 6.69 | 45.85 ± 9.05 | 43.18 ± 3.15 | 47.17 ± 6.51 | 52.92 ± 4.89 | <0.01 | 5.42 (-0.97 to 13.61) |
| adjusted | NA | NA | NA | 1.19 ± 1.17 | NA | 30.87 ± 4.40 | 45.70 ± 8.48 | 44.00 ± 3.59 | 47.72 ± 6.96 | 52.91 ± 4.62 | <0.01 | 6.17 (0.69 to 13.83) |
| **Chose low-calorie foods** |  |  |  |  |  |  |  |  |  |  |  |  |
| unadjusted | 16.70 ± 4.45 | 25.94 ± 5.11 | 28.70 ± 4.99 | 22.96 ± 3.71 | 35.49 ± 7.90 | 19.56 ± 5.92 | 21.89 ± 5.62 | 24.07 ± 3.99 | 18.04 ± 3.73 | 25.93 ± 4.55 | 0.81 | -0.34 (-3.48 to 2.68) |
| adjusted | 17.21 ± 4.63 | 26.17 ± 5.41 | 28.38 ± 5.30 | 23.37 ± 3.69 | 38.81 ± 7.45 | 17.73 ± 4.58 | 20.51 ± 5.19 | 24.24 ± 4.30 | 17.96 ± 3.44 | 25.24 ± 4.13 | 0.77 | -0.62 (-4.15 to 3.06) |
| **Ate less fat** |  |  |  |  |  |  |  |  |  |  |  |  |
| unadjusted | 23.55 ± 4.02 | 24.48 ± 3.93 | 31.24 ± 5.00 | 29.20 ± 4.25 | 26.97 ± 5.60 | 25.16 ± 5.62 | 22.62 ± 3.88 | 15.95 ± 2.72 | 22.44 ± 3.60 | 20.98 ± 3.12 | 0.03 | -1.73 (-3.58 to 0.12) |
| adjusted | 22.94 ± 3.94 | 24.88 ± 4.29 | 31.66 ± 4.96 | 29.43 ± 4.70 | 29.31 ± 5.26 | 24.30 ± 4.96 | 22.59 ± 3.62 | 15.53 ± 2.36 | 22.68 ± 3.51 | 21.81 ± 3.11 | 0.01 | -1.75 (-4.14 to 0.77) |
| **Skipped meals** |  |  |  |  |  |  |  |  |  |  |  |  |
| unadjusted | 24.72 ± 3.76 | 19.43 ± 2.74 | 24.28 ± 4.53 | 32.30 ± 4.59 | 23.98 ± 5.21 | 21.90 ± 4.65 | 13.55 ± 4.07 | 11.47 ± 3.66 | 22.47 ± 4.08 | 27.93 ± 4.22 | 0.77 | 0.03 (-3.45 to 2.87) |
| adjusted | 22.48 ± 2.83 | 19.39 ± 3.12 | 24.11 ± 4.67 | 32.58 ± 4.81 | 22.47 ± 4.85 | 20.90 ± 4.16 | 14.34 ± 3.96 | 11.09 ± 3.05 | 24.30 ± 3.28 | 28.26 ± 4.02 | 0.53 | 0.36 (-2.34 to 2.81) |
| **Ate less sugar, candy, sweets** |  |  |  |  |  |  |  |  |  |  |  |  |
| unadjusted | NA | NA | NA | 0.30 ± 0.31 | NA | 27.75 ± 6.07 | 32.90 ± 7.10 | 34.21 ± 2.84 | 35.98 ± 4.42 | 53.36 ± 5.12 | <0.01 | 10.04 (1.97 to 22.25) |
| adjusted | NA | NA | NA | 0.31 ± 0.31 | NA | 24.70 ± 4.14 | 31.79 ± 6.26 | 33.20 ± 2.72 | 35.74 ± 4.45 | 54.05 ± 5.01 | <0.01 | 10.81 (3.44 to 21.73) |
| **Changed eating habits** | NA | NA | NA |  | NA |  |  |  |  |  |  |  |
| unadjusted | NA | NA | NA | 1.56 ± 1.32 | NA | 32.19 ± 8.10 | 42.37 ± 6.73 | 37.49 ± 4.28 | 32.01 ± 3.81 | 38.78 ± 3.99 | <0.01 | 1.94 (-4.77 to 13.55) |
| adjusted | NA | NA | NA | 1.62 ± 1.22 | NA | 30.23 ± 6.34 | 43.15 ± 6.49 | 38.16 ± 4.05 | 34.03 ± 4.02 | 39.62 ± 4.07 | <0.01 | 2.73 (-4.23 to 13.79) |
| **Ate fewer carbohydrates** |  |  |  |  |  |  |  |  |  |  |  |  |
| unadjusted | NA | NA | NA | 14.86 ± 4.20 | 26.10 ± 6.90 | 5.52 ± 2.05 | 13.04 ± 5.57 | 12.44 ± 3.05 | 11.63 ± 2.63 | 17.59 ± 3.00 | 0.82 | -0.07 (-6.43 to 9.65) |
| adjusted | NA | NA | NA | 15.00 ± 4.04 | 27.10 ± 6.60 | 5.46 ± 1.87 | 12.17 ± 4.94 | 12.85 ± 3.44 | 11.82 ± 2.83 | 17.59 ± 3.09 | 0.81 | -0.34 (-6.75 to 8.41) |
| **Ate diet products** |  |  |  |  |  |  |  |  |  |  |  |  |
| unadjusted | 4.89 ± 2.41 | 10.95 ± 3.23 | 15.02 ± 4.07 | 19.55 ± 2.91 | 15.84 ± 6.59 | 7.03 ± 4.29 | 8.72 ± 4.18 | 8.11 ± 1.92 | 6.92 ± 3.19 | 14.50 ± 2.05 | 0.75 | -0.69 (-7.70 to 6.72) |
| adjusted | 4.34 ± 1.85 | 10.41 ± 3.11 | 14.94 ± 3.97 | 19.67 ± 2.77 | 13.86 ± 5.09 | 6.33 ± 3.35 | 8.52 ± 3.62 | 7.25 ± 1.47 | 6.16 ± 2.64 | 14.81 ± 2.34 | 0.71 | -0.91 (-8.35 to 6.70) |
| **Took non-prescription supplements** |  |  |  |  |  |  |  |  |  |  |  |  |
| unadjusted | 9.79 ± 3.57 | 8.49 ± 2.52 | 8.51 ± 3.40 | 9.71 ± 2.97 | 3.66 ± 2.25 | 7.89 ± 5.69 | 5.11 ± 1.81 | 8.36 ± 3.03 | 2.23 ± 1.72 | 6.39 ± 2.85 | 0.20 | -3.05 (-7.78 to 0.72) |
| adjusted | 10.39 ± 3.49 | 8.08 ± 2.71 | 7.08 ± 2.83 | 9.96 ± 2.92 | 3.93 ± 2.53 | 7.14 ± 4.88 | 5.66 ± 2.19 | 7.70 ± 2.86 | 2.85 ± 2.08 | 6.81 ± 2.94 | 0.22 | -2.72 (-8.18 to 1.56) |
| **Followed special diet** |  |  |  |  |  |  |  |  |  |  |  |  |
| unadjusted | 1.83 ± 1.49 | 4.63 ± 2.74 | 11.29 ± 2.61 | 7.32 ± 2.32 | 1.82 ± 1.09 | 0.82 ± 0.83 | 2.17 ± 1.10 | 7.30 ± 4.69 | 9.10 ± 5.08 | 6.15 ± 1.94 | 0.55 | -1.93 (-16.64 to 9.16) |
| adjusted | 1.79 ± 1.36 | 5.36 ± 2.91 | 9.95 ± 2.15 | 7.38 ± 2.47 | 2.19 ± 1.38 | 0.84 ± 0.82 | 2.01 ± 1.02 | 7.47 ± 4.32 | 8.26 ± 4.37 | 6.35 ± 1.88 | 0.65 | -1.41 (-13.57 to 7.82) |
| **Used liquid diet formula** |  |  |  |  |  |  |  |  |  |  |  |  |
| unadjusted | 6.88 ± 3.73 | 10.74 ± 3.60 | 9.94 ± 2.64 | 7.40 ± 2.69 | 4.22 ± 1.99 | 5.95 ± 2.32 | 5.37 ± 3.81 | 5.21 ± 2.90 | 0.73 ± 0.51 | 6.67 ± 2.10 | 0.08 | -4.17 (-10.36 to 0.75) |
| adjusted | 6.71 ± 3.37 | 11.99 ± 4.01 | 10.14 ± 2.46 | 7.63 ± 2.72 | 3.91 ± 1.80 | 6.24 ± 2.22 | 4.76 ± 2.73 | 4.61 ± 2.15 | 0.58 ± 0.36 | 6.81 ± 1.98 | 0.07 | -4.74 (-12.05 to 1.41) |
| **Joined weight loss program** |  |  |  |  |  |  |  |  |  |  |  |  |
| unadjusted | 2.78 ± 1.92 | NA | 8.43 ± 2.38 | 3.58 ± 2.17 | 3.75 ± 2.59 | 6.95 ± 4.31 | 1.46 ± 0.94 | 0.49 ± 0.50 | 3.37 ± 1.70 | 2.35 ± 1.26 | 0.05 | -6.90 (-21.36 to 5.46) |
| adjusted | 3.36 ± 2.28 | NA | 7.51 ± 2.37 | 3.51 ± 2.05 | 3.36 ± 2.20 | 6.61 ± 3.70 | 1.69 ± 1.37 | 0.59 ± 0.57 | 3.50 ± 1.67 | 2.50 ± 1.41 | 0.04 | -5.55 (-15.61 to 3.32) |
| **Other method** |  |  |  |  |  |  |  |  |  |  |  |  |
| unadjusted | 2.92 ± 1.58 | 6.60 ± 2.43 | 1.89 ± 1.25 | 0.27 ± 0.25 | 0.52 ± 0.52 | 1.27 ± 1.25 | 0.47 ± 0.49 | 0.64 ± 0.43 | 0.51 ± 0.53 | 1.75 ± 1.19 | 0.13 | -9.97 (-18.75 to -3.06) |
| adjusted | 3.13 ± 1.57 | 7.55 ± 2.83 | 2.24 ± 1.40 | 0.23 ± 0.20 | 0.90 ± 0.97 | 1.82 ± 1.76 | 0.42 ± 0.44 | 0.49 ± 0.32 | 0.41 ± 0.44 | 1.53 ± 1.06 | 0.17 | -11.38 (-20.95 to -3.90) |
| **Took prescribed diet pills** |  |  |  |  |  |  |  |  |  |  |  |  |
| unadjusted | 0.84 ± 0.83 | 3.34 ± 1.44 | 3.37 ± 1.83 | 2.54 ± 1.80 | 0.53 ± 0.50 | 2.85 ± 2.03 | 0.68 ± 0.71 | 1.97 ± 1.21 | 1.51 ± 1.20 | 0.93 ± 0.69 | 0.18 | -5.04 (-11.88 to 0.62) |
| adjusted | 1.12 ± 1.09 | 3.81 ± 1.78 | 3.15 ± 1.68 | 2.61 ± 1.85 | 0.49 ± 0.51 | 2.39 ± 1.65 | 0.90 ± 0.94 | 3.02 ± 1.61 | 2.13 ± 1.67 | 0.99 ± 0.74 | 0.19 | -3.57 (-12.37 to 4.25) |
| **Used laxatives or vomited** |  |  |  |  |  |  |  |  |  |  |  |  |
| unadjusted | 0.18 ± 0.18 | 4.84 ± 2.52 | 0.65 ± 0.29 | 0.52 ± 0.35 | 0.65 ± 0.65 | 1.88 ± 1.28 | 0.43 ± 0.44 | 0.61 ± 0.43 | 0.43 ± 0.43 | 0.89 ± 0.58 | 0.17 | -3.57 (-16.38 to 8.18) |
| adjusted | 0.13 ± 0.13 | 4.83 ± 2.66 | 0.69 ± 0.34 | 0.51 ± 0.32 | 0.51 ± 0.53 | 1.89 ± 1.23 | 0.38 ± 0.39 | 0.70 ± 0.52 | 0.34 ± 0.32 | 1.00 ± 0.67 | 0.15 | -2.84 (-14.23 to 7.93) |
| **Smoke** |  |  |  |  |  |  |  |  |  |  |  |  |
| unadjusted | NA | NA | NA | 0.39 ± 0.39 | 0.68 ± 0.70 | 0.83 ± 0.82 | 0.68 ± 0.71 | NA | 1.96 ± 1.65 | NA | 0.26 | NA |
| adjusted | NA | NA | NA | 0.43 ± 0.44 | 1.18 ± 1.26 | 0.62 ± 0.61 | 0.90 ± 0.94 | NA | 2.84 ± 2.30 | NA | 0.29 | NA |

NA denotes data unavailable for specific survey cycles. For most weight loss measures, the analyses were conducted from 1999 to March 2020; however, due to missing data in some cycles, analyses were performed only for those with available data. Proportions attempting specific weight loss measures are presented as weighted percentages ± standard error. Adjusted proportions are age-standardized using the 2000 U.S. Census population for adolescents aged 16-19 years. In the adjusted model, P for trend was additionally adjusted for age, sex, race/ethnicity, and family income. The average annual percent change is expressed as "estimate (95% confidence interval)" to represent the average yearly change over the study period.

**Table S9. Backward Elimination Multivariate Logistic Regression Analysis of the Association Between Weight Loss Measures and Successful Weight Loss**

| **Weight loss measures** | **Crude OR (95% CI)** | **P** | **Adjusted OR (95% CI)** | **P** |
| --- | --- | --- | --- | --- |
| **Weight Loss ≥5%** |  |  |  |  |
| Ate less food | 1.19 (0.90 to 1.57) | 0.22 | NA | NA |
| Ate less junk food or fast food | 0.77 (0.49 to 1.21) | 0.25 | 0.74 (0.46 to 1.20) | 0.19 |
| Ate more fruits, vegetables, salads | 0.69 (0.41 to 1.17) | 0.15 | 0.73 (0.45 to 1.19) | 0.23 |
| Ate less sugar, candy, sweets | 1.91 (1.14 to 3.20) | 0.01 | 2.16 (1.33 to 3.53) | <0.01 |
| Ate diet products | 0.48 (0.28 to 0.83) | <0.01 | 0.48 (0.28 to 0.83) | 0.01 |
| Used liquid diet formula | 0.63 (0.29 to 1.36) | 0.24 | 0.62 (0.30 to 1.34) | 0.22 |
| Took prescribed diet pills | 2.42 (1.02 to 5.75) | 0.04 | 2.55 (1.00 to 6.47) | <0.05 |
| **Weight Loss ≥10%** |  |  |  |  |
| Ate less food | 1.28 (0.88 to 1.69) | 0.21 | NA | NA |
| Ate less junk food or fast food | 0.69 (0.32 to 1.50) | 0.33 | 0.68 (0.31 to 1.48) | 0.31 |
| Ate more fruits, vegetables, salads | 0.63 (0.33 to 1.20) | 0.16 | 0.69 (0.37 to 1.30) | 0.26 |
| Chose low-calorie foods | 1.32 (0.80 to 2.16) | 0.29 | NA | NA |
| Ate less fat | NA | NA | 1.48 (0.88 to 2.49) | 0.14 |
| Ate less sugar, candy, sweets | 1.47 (0.69 to 3.14) | 0.33 | 1.80 (0.84 to 3.84) | 0.14 |
| Changed eating habits | 1.48 (0.78 to 2.83) | 0.23 | 1.51 (0.80 to 2.84) | 0.21 |
| Ate fewer carbohydrates | 1.68 (0.85 to 3.30) | 0.15 | 1.59 (0.81 to 3.12) | 0.19 |
| Ate diet products | 0.54 (0.28 to 1.03) | 0.09 | 0.54 (0.28 to 1.04) | 0.10 |
| Used liquid diet formula | 0.46 (0.12 to 1.49) | 0.27 | 0.46 (0.12 to 1.48) | 0.27 |
| Other method | 2.89 (0.99 to 8.46) | 0.06 | 2.70 (0.94 to 7.81) | 0.07 |
| Took prescribed diet pills | 3.12 (1.14 to 8.92) | 0.03 | 3.75 (1.37 to 10.31) | 0.01 |
| Smoke | 3.97 (0.39 to 40.67) | 0.25 | NA | NA |

Crude OR: Odds Ratio calculated with backward elimination applied to all weight loss measures.

Adjusted OR: Odds Ratio calculated with backward elimination applied to all weight loss measures, while adjusting for additional covariates such as age, sex, race, and family income.

Abbreviations: 95% CI: 95% Confidence Interval. OR: Odds Ratio. NA indicates the variable was not included in the model or was not applicable.

**Table S10. Multivariate Logistic Regression Analysis of the Association Between Weight Loss Measures and Successful Weight Loss**

| **Weight Loss Measures** | **Crude OR (95% CI)** | **P** | **Adjusted OR (95% CI)** | **P** |
| --- | --- | --- | --- | --- |
| **Weight Loss ≥5%** |  |  |  |  |
| Exercised | 1.15 (0.75 to 1.78) | 0.51 | 1.15 (0.74 to 1.81) | 0.53 |
| Ate less food | 1.22 (0.92 to 1.60) | 0.17 | 1.17 (0.86 to 1.59) | 0.31 |
| Drank a lot of water | 1.06 (0.76 to 1.63) | 0.75 | 1.04 (0.72 to 1.59) | 0.81 |
| Ate less junk food or fast food | 0.76 (0.49 to 1.22) | 0.22 | 0.72 (0.46 to 1.15) | 0.15 |
| Ate more fruits, vegetables, salads | 0.64 (0.39 to 1.06) | 0.09 | 0.67 (0.41 to 1.10) | 0.13 |
| Chose low-calorie foods | 1.25 (0.86 to 1.80) | 0.27 | 1.20 (0.83 to 1.74) | 0.39 |
| Ate less fat | 0.85 (0.57 to 1.44) | 0.46 | 0.94 (0.60 to 1.47) | 0.80 |
| Skipped meals | 0.84 (0.57 to 1.32) | 0.38 | 0.90 (0.60 to 1.37) | 0.62 |
| Ate less sugar, candy, sweets | 1.75 (1.02 to 3.00) | 0.04 | 1.95 (1.14 to 3.33) | 0.01 |
| Changed eating habits | 1.20 (0.76 to 1.94) | 0.43 | 1.25 (0.77 to 2.03) | 0.34 |
| Ate fewer carbohydrates | 0.99 (0.53 to 1.85) | 0.97 | 0.93 (0.51 to 1.68) | 0.81 |
| Ate diet products | 0.48 (0.28 to 0.82) | 0.01 | 0.48 (0.29 to 0.82) | 0.02 |
| Took non-prescription supplements | 0.94 (0.48 to 1.98) | 0.85 | 0.85 (0.44 to 1.86) | 0.63 |
| Followed special diet | 0.86 (0.40 to 1.99) | 0.71 | 0.81 (0.37 to 1.90) | 0.62 |
| Used liquid diet formula | 0.61 (0.22 to 1.63) | 0.23 | 0.60 (0.22 to 1.63) | 0.20 |
| Joined weight loss program | 0.97 (0.43 to 2.63) | 0.94 | 0.97 (0.39 to 2.55) | 0.95 |
| Other method | 1.42 (0.52 to 3.86) | 0.51 | 1.41 (0.50 to 3.98) | 0.53 |
| Took prescribed diet pills | 2.39 (0.94 to 5.83) | 0.07 | 2.53 (0.93 to 6.05) | 0.07 |
| Used laxatives or vomited | 1.42 (0.43 to 4.55) | 0.56 | 1.60 (0.48 to 5.12) | 0.45 |
| Smoke | 2.62 (0.33 to 8.87) | 0.38 | 1.91 (0.34 to 6.91) | 0.52 |
| **Weight Loss ≥10%** |  |  |  |  |
| Exercised | 1.00 (0.57 to 1.76) | 0.99 | 1.00 (0.59 to 1.67) | 0.98 |
| Ate less food | 1.31 (0.88 to 1.95) | 0.18 | 1.27 (0.87 to 1.87) | 0.25 |
| Drank a lot of water | 1.28 (0.81 to 2.02) | 0.29 | 1.29 (0.81 to 2.05) | 0.30 |
| Ate less junk food or fast food | 0.69 (0.32 to 1.47) | 0.33 | 0.68 (0.31 to 1.45) | 0.30 |
| Ate more fruits, vegetables, salads | 0.59 (0.30 to 1.14) | 0.12 | 0.63 (0.33 to 1.19) | 0.16 |
| Chose low-calorie foods | 1.27 (0.77 to 2.08) | 0.37 | 1.19 (0.70 to 2.00) | 0.53 |
| Ate less fat | 1.18 (0.71 to 1.96) | 0.53 | 1.34 (0.79 to 2.27) | 0.29 |
| Skipped meals | 0.78 (0.45 to 1.36) | 0.37 | 0.83 (0.48 to 1.44) | 0.49 |
| Ate less sugar, candy, sweets | 1.41 (0.66 to 3.02) | 0.37 | 1.54 (0.75 to 3.14) | 0.26 |
| Changed eating habits | 1.47 (0.78 to 2.78) | 0.24 | 1.53 (0.82 to 2.86) | 0.18 |
| Ate fewer carbohydrates | 1.55 (0.79 to 3.04) | 0.21 | 1.44 (0.76 to 2.75) | 0.28 |
| Ate diet products | 0.52 (0.27 to 1.04) | 0.07 | 0.52 (0.26 to 1.05) | 0.09 |
| Took non-prescription supplements | 0.83 (0.35 to 2.00) | 0.67 | 0.68 (0.26 to 1.78) | 0.42 |
| Followed special diet | 1.24 (0.53 to 2.92) | 0.62 | 1.11 (0.46 to 2.66) | 0.81 |
| Used liquid diet formula | 0.47 (0.11 to 1.59) | 0.30 | 0.46 (0.11 to 1.60) | 0.30 |
| Joined weight loss program | 0.65 (0.19 to 1.94) | 0.48 | 0.65 (0.20 to 1.93) | 0.49 |
| Other method | 2.92 (0.99 to 9.67) | 0.05 | 2.83 (0.94 to 8.88) | 0.07 |
| Took prescribed diet pills | 3.34 (1.13 to 9.04) | 0.02 | 3.59 (1.26 to 10.02) | 0.02 |
| Used laxatives or vomited | 1.57 (0.47 to 5.92) | 0.55 | 1.84 (0.51 to 7.01) | 0.43 |
| Smoke | 4.03 (0.28 to 10.69) | 0.27 | 2.53 (0.30 to 8.56) | 0.40 |

Note: Crude OR: Odds Ratio calculated with all weight loss measures included in the model. Adjusted OR: Odds Ratio calculated with all weight loss measures included in the model, while adjusting for covariates including age, sex, race, and family income.

Abbreviations: 95% CI: 95% Confidence Interval, OR: Odds ratios

**Table S11. Percentage of Participants Achieving** **≥0.2 Reduction in BMI z-score Among Those Attempting to Lose Weight, Stratified by Sex, Race, and BMI**

| **Stratification** | **Participants, No. (weighted %) (95% CI)** | | | **P-value** |
| --- | --- | --- | --- | --- |
|  | **Total (N=1640)** | **Boys (N=714)** | **Girls (N=** **926)** |  |
| Overall | 519 (34.34) (30.25–38.43) | 282 (40.28) (35.58–44.99) | 237 (29.30) (25.19–33.41) | <0.01 |
| By race |  |  |  | <0.01 |
| Non-Hispanic White | 159 (38.27) (32.89–43.65) | 81 (40.95) (33.42–48.47) | 78 (35.98) (28.99–42.98) |  |
| Non-Hispanic Black | 125 (27.83) (23.21–32.44) | 62 (41.57) (32.87–50.26) | 63 (20.74) (15.88–25.59) |  |
| Mexican American | 147 (30.86) (25.52–36.20) | 87 (40.67) (31.13–50.22) | 60 (21.04) (13.97–28.11) |  |
| Others | 88 (32.06) (26.10–38.01) | 52 (37.26) (28.21–46.32) | 36 (26.40) (18.75–34.05) |  |
| By BMI |  |  |  | <0.01 |
| Obesity | 293 (32.66) (28.72–36.59) | 181 (39.34) (33.39–45.29) | 112 (25.39) (20.41–30.36) |  |
| Overweight | 226 (36.81) (31.81–41.82) | 101 (42.25) (34.32–50.17) | 125 (33.65) (27.51–39.79) |  |

Abbreviations: CI, Confidence interval; BMI, body mass index.

**Table S12. Trends in ≥0.2 BMI z-score Reduction Among Adolescents (16-19 Years) Attempting Weight Loss, 1999-August 2023: Overall and by Sex and Weight Status**

| **Weight loss success rates** | **1999-2000** | **2001-2002** | **2003-2004** | **2005-2006** | **2007-2008** | **2009-2010** | **2011-2012** | **2013-2014** | **2015-2016** | **2017-March 2020** | **August 2021-August 2023** | **P for trend *** | **Average annual percent change (95% confidence interval)** |
| --- | --- | --- | --- | --- | --- | --- | --- | --- | --- | --- | --- | --- | --- |
| **All participants** |  |  |  |  |  |  |  |  |  |  |  |  |  |
| Unadjusted | 28.84 ± 5.30 | 33.99 ± 4.48 | 38.09 ± 5.49 | 36.62 ± 6.63 | 30.82 ± 7.02 | 47.28 ± 6.10 | 32.27 ± 5.21 | 24.85 ± 3.20 | 30.27 ± 4.69 | 33.51 ± 4.15 | 45.42 ± 3.81 | 0.50 | 0.01 (-2.33 to 2.48) |
| Adjusted | 27.14 ± 5.40 | 35.07 ± 5.01 | 38.20 ± 4.69 | 36.85 ± 7.08 | 32.85 ± 6.05 | 46.69 ± 4.94 | 32.41 ± 5.81 | 24.64 ± 3.65 | 30.76 ± 4.26 | 33.05 ± 4.05 | 44.32 ± 3.65 | 0.38 | 0.05 (-1.93 to 2.25) |
| **Overweight** |  |  |  |  |  |  |  |  |  |  |  |  | 0.04 (-3.16 to 3.63) |
| Unadjusted | 29.47 ± 10.52 | 40.21 ± 6.43 | 35.55 ± 6.20 | 48.73 ± 9.15 | 20.48 ± 8.50 | 41.40 ± 9.55 | 40.35 ± 12.01 | 19.29 ± 3.05 | 42.85 ± 6.57 | 32.73 ± 7.39 | 48.57 ± 7.14 | 0.65 | 0.25 (-2.45 to 3.23) |
| Adjusted | 27.37 ± 10.55 | 41.46 ± 6.93 | 38.21 ± 4.66 | 48.91 ± 9.25 | 24.21 ± 9.24 | 41.65 ± 9.09 | 37.18 ± 9.67 | 22.25 ± 4.49 | 45.60 ± 7.67 | 34.42 ± 7.59 | 48.14 ± 6.82 | 0.41 | 0.84 (-3.62 to 5.04) |
| **Obesity** |  |  |  |  |  |  |  |  |  |  |  |  | 0.61 (-4.78 to 5.31) |
| Unadjusted | 28.36 ± 6.83 | 26.35 ± 5.08 | 40.01 ± 8.63 | 26.20 ± 5.92 | 37.29 ± 7.88 | 49.77 ± 8.11 | 26.64 ± 7.05 | 27.62 ± 3.83 | 20.28 ± 6.06 | 33.95 ± 5.19 | 43.43 ± 5.67 | 0.54 | 0.07 (-3.70 to 3.69) |
| Adjusted | 26.95 ± 5.31 | 29.35 ± 6.02 | 38.51 ± 8.57 | 26.49 ± 6.35 | 38.13 ± 6.80 | 49.38 ± 5.82 | 27.00 ± 7.14 | 26.23 ± 3.86 | 21.68 ± 5.86 | 34.60 ± 4.82 | 42.04 ± 6.33 | 0.64 | 0.07 (-3.71 to 3.60) |
| **Boys** |  |  |  |  |  |  |  |  |  |  |  |  | -0.19 (-2.77 to 2.21) |
| Unadjusted | 30.90 ± 6.93 | 38.42 ± 6.65 | 40.90 ± 8.27 | 36.32 ± 8.63 | 46.53 ± 8.43 | 59.98 ± 9.45 | 40.88 ± 8.75 | 24.00 ± 5.30 | 33.39 ± 6.61 | 44.96 ± 7.64 | 50.98 ± 7.08 | 0.34 | -0.32 (-3.63 to 2.93) |
| Adjusted | 27.56 ± 6.11 | 39.75 ± 5.91 | 41.80 ± 7.43 | 37.53 ± 8.77 | 46.19 ± 9.46 | 58.33 ± 7.95 | 40.50 ± 8.33 | 21.86 ± 4.61 | 31.62 ± 7.42 | 44.56 ± 6.66 | 50.07 ± 6.56 | 0.33 | 0.14 (-3.41 to 3.95) |
| **Girls** |  |  |  |  |  |  |  |  |  |  |  |  | 0.72 (-2.92 to 5.33) |
| Unadjusted | 27.60 ± 6.64 | 30.56 ± 5.03 | 34.70 ± 5.17 | 36.81 ± 7.06 | 19.17 ± 7.31 | 36.79 ± 5.59 | 24.85 ± 8.36 | 25.93 ± 6.31 | 28.38 ± 6.45 | 24.18 ± 5.01 | 39.23 ± 10.05 | 0.87 | -0.12 (-7.85 to 6.79) |
| Adjusted | 25.70 ± 6.77 | 31.09 ± 5.33 | 33.94 ± 5.15 | 38.02 ± 7.06 | 19.88 ± 5.16 | 34.27 ± 4.68 | 20.50 ± 6.64 | 25.50 ± 6.79 | 30.26 ± 5.99 | 23.57 ± 4.67 | 32.71 ± 7.09 | 0.90 | -1.08 (-10.90 to 7.82) |
| **Boys Overweight** |  |  |  |  |  |  |  |  |  |  |  |  | 0.55 (-2.76 to 4.09) |
| Unadjusted | 32.57 ± 12.10 | 36.70 ± 11.50 | 44.17 ± 11.05 | 50.15 ± 13.29 | 43.98 ± 13.08 | 73.61 ± 12.37 | 37.01 ± 13.71 | 24.30 ± 8.08 | 48.19 ± 14.15 | 39.42 ± 13.16 | 55.07 ± 10.53 | 0.69 | 0.07 (-4.17 to 4.61) |
| Adjusted | 42.90 ± 7.99 | 30.66 ± 7.57 | 50.04 ± 4.81 | 50.46 ± 11.31 | 41.94 ± 11.63 | 72.05 ± 9.25 | 39.16 ± 10.34 | 23.72 ± 9.49 | 41.98 ± 8.28 | 42.28 ± 6.76 | 48.58 ± 9.61 | 0.49 | 0.69 (-6.73 to 8.17) |
| **Boys Obesity** |  |  |  |  |  |  |  |  |  |  |  |  | -0.26 (-4.95 to 4.08) |
| Unadjusted | 29.93 ± 10.00 | 40.25 ± 7.66 | 38.24 ± 9.67 | 28.59 ± 6.96 | 47.74 ± 11.49 | 56.67 ± 11.77 | 42.36 ± 11.40 | 23.91 ± 5.38 | 25.65 ± 11.55 | 47.41 ± 9.00 | 49.66 ± 9.65 | 0.33 | 0.01 (-2.33 to 2.48) |
| Adjusted | 29.66 ± 7.35 | 48.83 ± 8.39 | 34.27 ± 10.05 | 28.76 ± 6.61 | 47.77 ± 10.96 | 50.63 ± 9.86 | 51.08 ± 10.11 | 21.79 ± 4.08 | 27.66 ± 9.87 | 46.55 ± 7.28 | 50.74 ± 9.12 | 0.28 | 0.05 (-1.93 to 2.25) |
| **Girls Overweight** |  |  |  |  |  |  |  |  |  |  |  |  | 0.04 (-3.16 to 3.63) |
| Unadjusted | 28.02 ± 13.29 | 42.61 ± 6.70 | 24.18 ± 7.84 | 48.11 ± 10.36 | 7.45 ± 3.71 | 27.80 ± 8.92 | 41.87 ± 15.46 | 15.56 ± 5.40 | 40.64 ± 7.81 | 28.60 ± 9.09 | 45.34 ± 9.85 | 0.72 | 0.25 (-2.45 to 3.23) |
| Adjusted | 27.32 ± 14.19 | 43.74 ± 7.46 | 27.65 ± 6.62 | 48.49 ± 10.62 | 12.69 ± 6.29 | 26.72 ± 7.11 | 32.69 ± 10.68 | 21.08 ± 4.93 | 43.24 ± 7.67 | 26.82 ± 7.70 | 41.00 ± 8.74 | 0.45 | 0.84 (-3.62 to 5.04) |
| **Girls Obesity** |  |  |  |  |  |  |  |  |  |  |  |  | 0.61 (-4.78 to 5.31) |
| Unadjusted | 27.21 ± 7.90 | 13.88 ± 3.83 | 42.01 ± 11.05 | 24.08 ± 7.16 | 28.05 ± 8.46 | 42.34 ± 8.25 | 5.91 ± 3.70 | 33.87 ± 7.28 | 15.99 ± 6.62 | 21.19 ± 4.88 | 31.86 ± 15.18 | 0.57 | 0.07 (-3.70 to 3.69) |
| Adjusted | 24.93 ± 6.47 | 16.78 ± 4.60 | 39.70 ± 10.20 | 27.03 ± 7.60 | 27.00 ± 5.40 | 36.77 ± 6.53 | 5.64 ± 3.07 | 32.74 ± 7.77 | 18.95 ± 5.10 | 22.32 ± 5.08 | 24.55 ± 10.58 | 0.72 | 0.07 (-3.71 to 3.60) |

Participation rates are presented as weighted percentages ± standard error. Adjusted rates are age-standardized using the 2000 U.S. Census population for adolescents aged 16-19 years. In the adjusted model, P for trend was additionally adjusted for age, sex, race/ethnicity, and family income. The average annual percent change is expressed as "estimate (95% confidence interval)" to represent the average yearly change over the study period.

**Table S13. Multivariate Logistic Regression Analysis of the Association Between Weight Loss Measures and Successful Weight Loss (≥0.2 BMI z-score Reduction)**

| **Weight loss measures** | **Crude OR (95% CI)** | **P** | **Adjusted OR (95% CI)** | **P** |
| --- | --- | --- | --- | --- |
| **Backward Elimination Multivariate Logistic Regression** |  |  |  |  |
| Ate less junk food or fast food | 0.79 (0.53 to 1.17) | 0.23 | 0.76 (0.51 to 1.12) | 0.17 |
| Chose low-calorie foods | 1.27 (0.85 to 1.91) | 0.25 | 1.28 (0.84 to 1.97) | 0.26 |
| Ate less sugar, candy, sweets | 1.76 (1.20 to 2.59) | <0.01 | 1.97 (1.33 to 2.91) | <0.01 |
| Ate fewer carbohydrates | 0.74 (0.41 to 1.31) | 0.30 | 0.74 (0.41 to 1.33) | 0.31 |
| Followed special diet | 0.68 (0.33 to 1.43) | 0.31 | 0.67 (0.30 to 1.47) | 0.32 |
| Used liquid diet formula | 0.48 (0.23 to 0.99) | 0.05 | 0.50 (0.25 to 1.07) | 0.08 |
| Other method | 2.34 (0.96 to 5.69) | 0.06 | 2.61 (1.02 to 6.67) | 0.05 |
| Took prescribed diet pills | 2.29 (0.96 to 5.45) | 0.06 | 2.87 (1.15 to 7.19) | 0.03 |
| **Full Multivariate Logistic Regression** |  |  |  |  |
| Exercised | 1.02 (0.70 to 1.48) | 0.91 | 0.99 (0.68 to 1.44) | 0.94 |
| Ate less food | 1.10 (0.81 to 1.50) | 0.54 | 1.08 (0.78 to 1.49) | 0.65 |
| Drank a lot of water | 1.02 (0.74 to 1.43) | 0.89 | 1.01 (0.71 to 1.45) | 0.94 |
| Ate less junk food or fast food | 0.79 (0.52 to 1.19) | 0.26 | 0.74 (0.49 to 1.11) | 0.15 |
| Ate more fruits, vegetables, salads | 0.87 (0.55 to 1.38) | 0.56 | 0.95 (0.60 to 1.51) | 0.83 |
| Chose low-calorie foods | 1.29 (0.84 to 1.99) | 0.25 | 1.28 (0.81 to 2.02) | 0.29 |
| Ate less fat | 1.11 (0.75 to 1.63) | 0.61 | 1.17 (0.78 to 1.75) | 0.44 |
| Skipped meals | 0.83 (0.59 to 1.17) | 0.28 | 0.87 (0.61 to 1.23) | 0.42 |
| Ate less sugar, candy, sweets | 1.76 (1.09 to 2.86) | 0.02 | 1.89 (1.16 to 3.08) | 0.01 |
| Changed eating habits | 1.16 (0.74 to 1.84) | 0.52 | 1.24 (0.78 to 1.98) | 0.37 |
| Ate fewer carbohydrates | 0.73 (0.41 to 1.28) | 0.27 | 0.72 (0.41 to 1.27) | 0.26 |
| Ate diet products | 0.76 (0.48 to 1.21) | 0.25 | 0.77 (0.47 to 1.26) | 0.29 |
| Took non-prescription supplements | 0.85 (0.45 to 1.61) | 0.62 | 0.84 (0.45 to 1.59) | 0.61 |
| Followed special diet | 0.72 (0.34 to 1.50) | 0.38 | 0.69 (0.30 to 1.55) | 0.36 |
| Used liquid diet formula | 0.53 (0.24 to 1.12) | 0.10 | 0.55 (0.26 to 1.15) | 0.11 |
| Joined weight loss program | 1.21 (0.51 to 2.87) | 0.66 | 1.35 (0.55 to 3.37) | 0.51 |
| Other method | 2.32 (0.93 to 5.80) | 0.07 | 2.58 (0.99 to 6.75) | 0.06 |
| Took prescribed diet pills | 2.29 (0.92 to 5.73) | 0.08 | 2.73 (1.02 to 7.32) | <0.05 |
| Used laxatives or vomited | 1.01 (0.31 to 3.29) | 0.99 | 1.25 (0.38 to 4.09) | 0.71 |
| Smoke | 1.58 (0.22 to 11.40) | 0.65 | 1.53 (0.25 to 9.06) | 0.64 |

Successful weight loss was defined as a ≥0.2 BMI z-score reduction.

Backward elimination model starts with all weight loss measures and sequentially deletes the least contributive measures for an optimal model; full model includes all weight loss measures without elimination. Crude ORs were unadjusted, and adjusted ORs were adjusted for age, sex, race and family income.

Abbreviations: 95% CI: 95% Confidence Interval, OR: Odds ratios
